# Supplementary material for: Wealth, health, and happiness: An inverse story of the Easterlin Paradox in China
Source: PLoS One. 2026 Mar 12;21(3):e0342445. doi: 10.1371/journal.pone.0342445 (PMC12981521; doi:10.1371/journal.pone.0342445)
Supplement: S1 Appendix — (DOCX) [file pone.0342445.s001.docx]

**Appendix A. Additional Results without the** $\boldsymbol{P}\boldsymbol{\times}\boldsymbol{f}\left( \boldsymbol{t} \right)$ **Interaction Term**

This appendix reports regression results and parallel-trend tests from specifications that exclude the interaction term $P\times f\left( t \right)$. These results are provided for comparison with the baseline models in the main text, which include $P\times f\left( t \right)$ to account for potential differential pre-policy trends associated with initial pollution levels.

Table A1. The impact of the TCZ Policy on SO_2_

|  | (1) | (2) |
| --- | --- | --- |
| VARIABLES | SO_2_ | SO_2_ |
|  |  |  |
| TCZ×Post | 0.385 | 0.473 |
|  | (0.334) | (0.337) |
| Rain |  | -0.001 |
|  |  | (0.000) |
| Temperature |  | -0.664* |
|  |  | (0.382) |
| Wind speed |  | -1.503** |
|  |  | (0.687) |
|  |  |  |
| Year FE | YES | YES |
| City FE | YES | YES |
| Province-by-year FE | YES | YES |
| Observations | 745 | 745 |
| R-squared | 0.940 | 0.940 |

Note: Table A1 reports the impact of the TCZ policy on city-level SO₂ emissions using specifications that exclude the interaction term $P\times f(t)$. All regressions include city fixed effects, year fixed effects, and province-by-year fixed effects. Robust standard errors are reported in parentheses (*** p<0.01, ** p<0.05, * p<0.1).

Table A2. Impact of the TCZ Policy on log monthly income

|  | (1) | (2) | (3) |
| --- | --- | --- | --- |
| VARIABLES | Ln(R) | Ln(R) | Ln(R) |
|  |  |  |  |
| TCZ×Post | -0.035 | -0.056** | -0.061*** |
|  | (0.025) | (0.023) | (0.023) |
| Education |  | 0.006 | 0.006 |
|  |  | (0.006) | (0.006) |
| Age |  | 0.096 | 0.092 |
|  |  | (0.069) | (0.069) |
| Age² |  | -0.050*** | -0.050*** |
|  |  | (0.007) | (0.007) |
| Married |  | 0.045* | 0.045* |
|  |  | (0.026) | (0.026) |
| Divorce |  | 0.152** | 0.152** |
|  |  | (0.059) | (0.059) |
| Widowed |  | 0.100 | 0.098 |
|  |  | (0.081) | (0.081) |
| Household income |  | 0.008*** | 0.008*** |
|  |  | (0.001) | (0.001) |
| Household members |  | -0.027*** | -0.026*** |
|  |  | (0.008) | (0.008) |
| Rain |  |  | 0.000* |
|  |  |  | (0.000) |
| Temperature |  |  | 0.029 |
|  |  |  | (0.024) |
| Wind speed |  |  | 0.001 |
|  |  |  | (0.036) |
|  | (85.729) | (80.392) | (88.977) |
|  |  |  |  |
| Individual FE | YES | YES | YES |
| Year FE | YES | YES | YES |
| City FE | YES | YES | YES |
| Province-by-year FE | YES | YES | YES |
| Observations | 11,929 | 11,088 | 11,088 |
| R-squared | 0.750 | 0.799 | 0.799 |

Note: Table A2 reports the impact of the TCZ policy on log income using specifications that exclude the interaction term $P\times f(t)$. All regressions include city fixed effects, year fixed effects, and province-by-year fixed effects. Robust standard errors are reported in parentheses (*** p<0.01, ** p<0.05, * p<0.1).


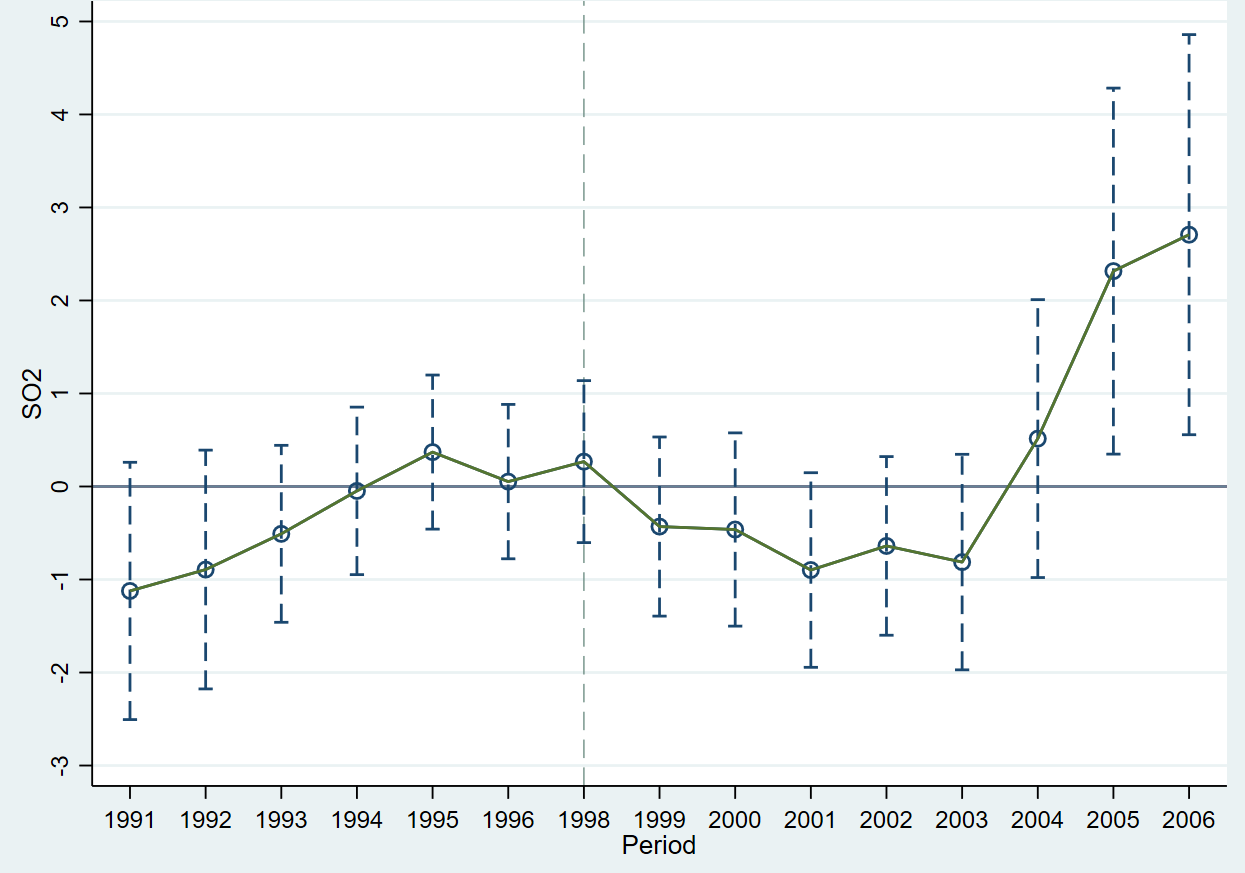


**Figure A1**. Parallel Trend Test (the TCZ on SO_2_).

Note: The baseline year is 1997, and the policy implementation year is 1998.


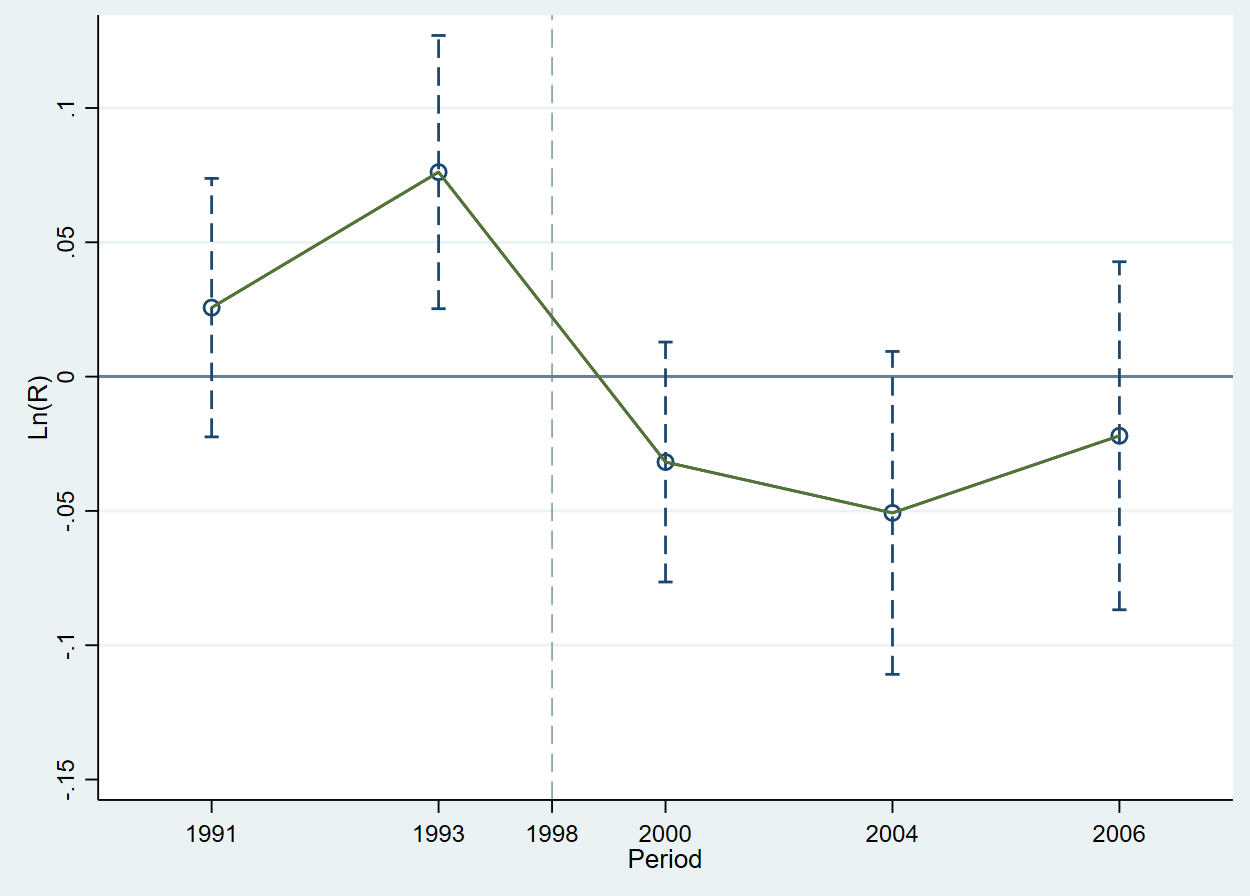


**Figure A2.** Parallel Trend Test (the TCZ on log monthly income).

Note: The baseline year is 1997, and the policy implementation year is 1998.

**Appendix B. Balancing Tests for Time-Varying Covariates**

To further examine whether TCZ policy can be regarded as plausibly exogenous with respect to time-varying covariates, we conduct a series of balancing tests following Tanaka [1] and Wang et al. [2].

Specifically, we regress the TCZ indicator on individual, household, and local weather characteristics. Column (1) of Table B1 reports results without fixed effects, while Column (2) includes individual, year, city, and province-by-year fixed effects, as well as the interaction term $P\times f\left( t \right)$.

After controlling for fixed effects and differential time trends, most coefficients become statistically insignificant, suggesting that the TCZ policy is not systematically correlated with observable time-varying characteristics. This provides descriptive evidence that observable time-varying covariates do not exhibit strong differential changes associated with TCZ policy.

Table B1. Balance test

| VARIABLES | (1) | (2) |
| --- | --- | --- |
| Education | 0.010*** | 0.001 |
|  | (0.000) | (0.003) |
| Age | 0.003*** | -0.122*** |
|  | (0.000) | (0.041) |
| Age² | 0.003*** | 0.002 |
|  | (0.000) | (0.004) |
| Marriage | 0.047*** | -0.003 |
|  | (0.002) | (0.012) |
| Household income | 0.002*** | 0.000 |
|  | (0.000) | (0.000) |
| Household members | -0.038*** | -0.000 |
|  | (0.002) | (0.006) |
| Rain | -0.026 | 0.012 |
|  | (0.034) | (0.050) |
| Temperature | 0.007** | 0.033 |
|  | (0.003) | (0.032) |
| Wind speed | 0.062*** | 0.106* |
|  | (0.019) | (0.059) |
| P×Year dummy | NO | YES |
| Individual FE | NO | YES |
| Year FE | NO | YES |
| City FE | NO | YES |
| Province-by-year FE | NO | YES |

Note: Table B1 presents regressions of the TCZ on control variables. Column (1) presents the specification without fixed effects, while Column (2) includes fixed effects and $P\times f(t)$. Robust standard errors are reported in parentheses (*** p<0.01, ** p<0.05, * p<0.1).

**References**

1. Tanaka, S. (2015). Environmental regulations on air pollution in China and their impact on infant mortality. Journal of Health Economics, 42, 90 - 103.
2. Wang, Y., Sun, C., Wang, J., Mao, X. (2024). Do People Feel Healthier After Holding Sports Mega-Events? Evidence from the 2010 Guangzhou Asian Games. Journal of Sports Economics, 25(5), 583-609.
