# Supplementary material for: Wealth, health, and happiness: An inverse story of the Easterlin Paradox in China
Source: PLoS One. 2026 Mar 12;21(3):e0342445. doi: 10.1371/journal.pone.0342445 (PMC12981521; doi:10.1371/journal.pone.0342445)
Supplement: Attachment — Submitted filename: Response to Reviewers.docx [file pone.0342445.s002.docx]

Dear editor,

Thank you for your email of 18 September 2024 concerning our manuscript “Wealth, Health, and Happiness: An Inverse Story of the Easterlin Paradox in China” (PONE-D-23-32960). We would like to thank you for the full consideration of our paper and sending us the comments of two reviewers. We have revised the paper according to yours and the reviewers’ suggestions, and mark the changed text in red.

The major revisions are summarized as follows:

1. We made a series of modifications to the methodology used in our research, specifically:

1) We no longer predict SWB across datasets, instead, we adopted the three steps approach suggested by you to evaluate the effect of TCZ policy.

2) We explore more details about how WTP changes over time and use the knowledge to provide robustness checks regarding the concern about period difference across data sets.

3) We used average log wage for staffs who work in the same industry and the same province as instrument variables for income.

2.	We made a series of modifications in the Literature review and Background components, including:

1) We placed the literature review before the background as suggested.

2) We deleted several less relevant contents from these two parts.

3) We added several important literatures as suggested.

3.	We had a professional editing of the paper. Now the writing has been substantially improved.

With this letter, we have resubmitted our manuscript which we would like to be considered for publication in Plos one. In order to facilitate the review of our revisions, we are attaching a detailed, comment by comment response to the two reviewers’ concerns.

If you need anything else, please do not hesitate to contact us.

Kind regards,

All authors

Response of Authors to the Comments of Editor

“Wealth, Health, and Happiness: An Inverse Story of the Easterlin Paradox in China”

| **Editor’s Comments** | **Authors’ Response** |
| --- | --- |
| 1. The topic is interesting and you have  done a lot of work, but using CHIPS data's SWB information to predict SWB in  CHNS is problematic. Actually, you do not have to do so. I have the following  proposal to answer your research question without imputing SWB:   - estimate the effect of two-control zone policy on air pollution, wage, and health, this can be done using only CHNS; - estimate the trade off between pollution (or health) and income, using only CHIPS, following Levison (2012). - Combining both 1 and 2, you can still back up the net effect of (net willingness to pay for) the two-control zone policy, as you did in Table 8. - You can use PSM method to predict individual's SWB in CHNS without inflating the sample size, and check if the effect for TCZ policy on imputed SWB matches your calculation in step 3, if you wish. But I don't think this is a must. | Thank you for your valuable suggestions. That provides us with a good framework to address reviewers’ key concerns. As suggested, we now choose to use a three-stage approach to combine information from CHNS data and CHIP data and to evaluate the total effect of TCZ policy on SWB. “First, we use a typical difference in difference (DID) approach to estimate the effect of the TCZ policy on air pollution and residential income of affected cities. For air pollution, we regress: \| $P_{jpt}=\alpha_{1}+\beta_{1}\left( {TCZ}_{j}\times{Post}_{t} \right)+\varphi_{1}W_{jpt}+\delta_{j}+\theta_{t}+\mu_{pt}+\varepsilon_{jt}$ \| (1) Here, $P_{jpt}$ is the air pollution of city $j$ (which locates in province $p$) at time $t$, measured by average ambient concentration of SO_2_ at the city level. ${TCZ}_{j}$ is a dummy variable indicating whether the observation is from a city covered by the TCZ policy (takes the value of 1 if it is) while ${Post}_{t}$ is a dummy variable indicating whether the observation is from the post-policy period (takes the value of 1 in the year 1998 and so after). $\hat{\beta}_{1}$ is then the parameter of interest which illustrates the effect of TCZ policy on city air pollution. In this regression, we control a series of city level meteorological factors and economic factors such as temperature, precipitation and logarithm of local GDP, the city fixed effect $\delta_{j}$, the time fixed effect $\theta_{t}$, and the combined fixed effect at a higher dimension, i.e., $\mu_{pt}={Province}_{j}\times{time}_{t}$. $\varepsilon_{jpt}$ is a heteroskedasticity-robust standard error term. For residential income, we regress: \| $R_{ihcjpt}=\alpha_{2}+\beta_{2}\left( {TCZ}_{j}\times{Post}_{t} \right)+\eta_{2}X_{ihcjpt}+\phi_{2}M_{chjpt}+\nu_{2}D_{cjpt}+\varphi_{2}W_{jpt}+\delta_{j}+\theta_{t}+\mu_{pt}+\varepsilon_{ihcjpt}$ \| (2) Here, $R_{ihcjpt}$ is the average monthly income of individual $i$ at time $t$, where the individual is from household $h$ and lives in the community $c$ of city $j$ in province $p$. $X_{ihcjpt}$ is a series of individual level control variables including the education year, gender, ethnicity and age. $M_{chjpt}$ is a series of household level control variables including family size and the household’s income. $D_{cjpt}$ is a series of community level control variables including the population density and the market composition index. $W_{jpt}$, $\delta_{j}$, $\theta_{t}$ and $\mu_{pt}$ are the same as in equation (1), and $\hat{\beta}_{2}$ is the parameter of interest which indicates the effect of TCZ policy on monthly income of affected individuals. The above two regressions are estimated using the CHNS data. Second, following the wisdom of Levinson (2012), Ambrey et al. (2014) and Zhang et al. (2017a, 2017b), we estimate residents’ WTP for improved air quality by exploring their trade-offs between economic benefit and environmental quality subject to the constraint that their SWB stay the same. Since both air quality and income might be correlated with factors which also influence SWB, we apply a series of instrumental variables to deal with potential endogeneity issues. We also use the methods adopted by the ordered choice models to take care of the potential problems from the discrete choice issues following Dolan et al. (2008), Diener et al. (2018) and Clark (2018).” “We use a 2SLS approach to estimate this IV model. The 1^st^ stage regressions are: \| $P_{jpt}=\alpha_{3}+\beta_{3}{Days}_{jpt}+\gamma_{3}{Wind}_{jpt}+\varphi_{3}W_{jpt}+\delta_{j}+\theta_{t}+\mu_{pt}+\varepsilon_{jt}$ \| (3) and: \| $lnR_{ihjkpt}=\alpha_{4}+\beta_{4}ln{Wage}_{kpt}+\eta_{4}X_{ihjt}+\phi_{4}M_{hjpt}+\varphi_{4}W_{jpt}+\delta_{j}+\theta_{t}+\mu_{pt}+\varepsilon_{ihjpt}$ \| (4) In equation (3), ${Days}_{jpt}$ is the weighted average of number of days in the year when the second layer of the atmosphere is warmer than the first layer and that when the third layer of the atmosphere is warmer than the first layer. The weight is 1:1. ${Wind}_{jpt}$ is the annual average wind speed of the city. In equation (4), $lnR_{ihjkpt}$ is the log income of individual $i$ at time $t$, where individual $i$ is from household $h$, lives in the community $c$ of city $j$ in province $p$, and works for industry $k$. $ln{Wage}_{kpt}$ is the average log wage for staffs who work in the same industry and the same province as individual $i$. Other control variables are the same as in equation (1) and (2). In the 2^nd^ stage, we regress the following ordered choice model: \| ${SWB}_{ihjkpt}=\alpha_{5}+\beta_{5}\hat{P}_{jpt}+\gamma_{5}\ln\hat{R}_{ihjkpt}+\eta_{5}X_{ihjpt}+\phi_{5}M_{hjpt}+\varphi_{5}W_{jpt}+\delta_{j}+\theta_{t}+\mu_{pt}+\varepsilon_{ihjpt}$ \| (5) Here, $\hat{P}_{jpt}$ are predicted through equation (3) while $\ln\hat{R}_{ihjkpt}$ are predicted from equation (4).” “according to Levinson (2012), Ambrey et al. (2014) and Zhang et al. (2017a, 2017b), the WTP of affected residents on improved air quality (measured by reduced SO_2_ concentration) can be calculated by: $\hat{WTP}=\frac{\partial R}{\partial P}\left\vert\begin{matrix} \\ \\ dSWB=0 \end{matrix} \right.=-\bar{R}\frac{\hat{\beta}_{5}}{\hat{\gamma}_{5}}$ Here, $\bar{R}$ is the average income of the sample. Equation (3)~(7) are estimated using the CHIP data. Finally, with influence of TCZ policy on air pollution ($\hat{\beta}_{1}$) and monthly income of affected residents ($\hat{\beta}_{2}$) estimated in the first step and the residents’ WTP for less air pollution estimated in the second step, we can calculate the average net money value of TCZ policy on affected residents through: $Value\left( \Delta SWB \right)=\hat{\beta}_{1}\hat{WTP}+\hat{\beta}_{2}$” The related content can be found in page 22-27 of our replenished manuscript. |

Response of Authors to the Comments of Reviewer #1

“Wealth, Health, and Happiness: An Inverse Story of the Easterlin Paradox in China”

| **Reviewer’s Comments** | **Authors’ Response** |
| --- | --- |
| Comments to the Author  GENERAL COMMENTS  This paper proposes the environmental explanation to the Easterlin paradox. Using the survey data of CHNS and CHIP, the paper estimates the effect of Tow Control Zone policy on households’ subjective wellbeing with a difference-in-difference approach, and further calculate the households’ willingness to pay for the environment. In general, I feel that the paper is well-written, the ideas is clear and easy to follow. Yet, there are some concerns regarding the research design and the empirical approach that need to be addressed to pass on to the next step. Following are some of my comments. | Thank you for all your nice comments and suggestions. Through the revisions, this paper has been substantially improved. We have revised the paper according to your comments. We hope you would find the revisions satisfactory. |
| MAJOR COMMENTS  1) Prediction of household subjective wellbeing (SWB). To derive the dependent variable of SWB, which is absent in the CHNS data, the authors construct a SWB function (model 1) to predict the SWB probability using another CHIP data. A key assumption underlying the approach is that the estimated SWB function is national representative, so that it can be transferred from one data to another. However, given the fact that the two datasets have different geographical coverage and time periods, it is hard for me to believe that households in the two datasets have the same preferences, and that you can transfer the SWB function from one group of households to another group of households. You should further justify the representativeness of the CHIP data and the appropriateness of your approach. | Thank you for highlighting the potential issues regarding the use of CHIP data to construct the SWB function and its application to CHNS data. We appreciate your valuable suggestions, and we agree that trying to combine information from two data sets with different geographic areas and periods is challenging. After carefully discussions around opinions from the editor and two reviewers, we have made following changes to our empirical strategy in identifying the comprehensive effects of TCZ policy on SWB. First, we no longer trying to predict the SWB for CHNS interviewers using CHIP data. Instead, following the suggestion from the editor, we use a less complex approach to draw information from CHIP data and CHNS data. The main process are as follows: “First, we use a typical difference in difference (DID) approach to estimate the effect of the TCZ policy on air pollution and residential income of affected cities. For air pollution, we regress: \| $P_{jpt}=\alpha_{1}+\beta_{1}\left( {TCZ}_{j}\times{Post}_{t} \right)+\varphi_{1}W_{jpt}+\delta_{j}+\theta_{t}+\mu_{pt}+\varepsilon_{jt}$ \| (1) Here, $P_{jpt}$ is the air pollution of city $j$ (which locates in province $p$) at time $t$, measured by average ambient concentration of SO_2_ at the city level. ${TCZ}_{j}$ is a dummy variable indicating whether the observation is from a city covered by the TCZ policy (takes the value of 1 if it is) while ${Post}_{t}$ is a dummy variable indicating whether the observation is from the post-policy period (takes the value of 1 in the year 1998 and so after). $\hat{\beta}_{1}$ is then the parameter of interest which illustrates the effect of TCZ policy on city air pollution. In this regression, we control a series of city level meteorological factors and economic factors such as temperature, precipitation and logarithm of local GDP, the city fixed effect $\delta_{j}$, the time fixed effect $\theta_{t}$, and the combined fixed effect at a higher dimension, i.e., $\mu_{pt}={Province}_{j}\times{time}_{t}$. $\varepsilon_{jpt}$ is a heteroskedasticity-robust standard error term. For residential income, we regress: \| $R_{ihcjpt}=\alpha_{2}+\beta_{2}\left( {TCZ}_{j}\times{Post}_{t} \right)+\eta_{2}X_{ihcjpt}+\phi_{2}M_{chjpt}+\nu_{2}D_{cjpt}+\varphi_{2}W_{jpt}+\delta_{j}+\theta_{t}+\mu_{pt}+\varepsilon_{ihcjpt}$ \| (2) Here, $R_{ihcjpt}$ is the average monthly income of individual $i$ at time $t$, where the individual is from household $h$ and lives in the community $c$ of city $j$ in province $p$. $X_{ihcjpt}$ is a series of individual level control variables including the education year, gender, ethnicity and age. $M_{chjpt}$ is a series of household level control variables including family size and the household’s income. $D_{cjpt}$ is a series of community level control variables including the population density and the market composition index. $W_{jpt}$, $\delta_{j}$, $\theta_{t}$ and $\mu_{pt}$ are the same as in equation (1), and $\hat{\beta}_{2}$ is the parameter of interest which indicates the effect of TCZ policy on monthly income of affected individuals. The above two regressions are estimated using the CHNS data. Second, following the wisdom of Levinson (2012), Ambrey et al. (2014) and Zhang et al. (2017a, 2017b), we estimate residents’ WTP for improved air quality by exploring their trade-offs between economic benefit and environmental quality subject to the constraint that their SWB stay the same. Since both air quality and income might be correlated with factors which also influence SWB, we apply a series of instrumental variables to deal with potential endogeneity issues. We also use the methods adopted by the ordered choice models to take care of the potential problems from the discrete choice issues following Dolan et al. (2008), Diener et al. (2018) and Clark (2018).” “We use a 2SLS approach to estimate this IV model. The 1^st^ stage regressions are: \| $P_{jpt}=\alpha_{3}+\beta_{3}{Days}_{jpt}+\gamma_{3}{Wind}_{jpt}+\varphi_{3}W_{jpt}+\delta_{j}+\theta_{t}+\mu_{pt}+\varepsilon_{jt}$ \| (3) and: \| $lnR_{ihjkpt}=\alpha_{4}+\beta_{4}ln{Wage}_{kpt}+\eta_{4}X_{ihjt}+\phi_{4}M_{hjpt}+\varphi_{4}W_{jpt}+\delta_{j}+\theta_{t}+\mu_{pt}+\varepsilon_{ihjpt}$ \| (4) In equation (3), ${Days}_{jpt}$ is the weighted average of number of days in the year when the second layer of the atmosphere is warmer than the first layer and that when the third layer of the atmosphere is warmer than the first layer. The weight is 1:1. ${Wind}_{jpt}$ is the annual average wind speed of the city. In equation (4), $lnR_{ihjkpt}$ is the log income of individual $i$ at time $t$, where individual $i$ is from household $h$, lives in the community $c$ of city $j$ in province $p$, and works for industry $k$. $ln{Wage}_{kpt}$ is the average log wage for staffs who work in the same industry and the same province as individual $i$. Other control variables are the same as in equation (1) and (2). In the 2^nd^ stage, we regress the following ordered choice model: \| ${SWB}_{ihjkpt}=\alpha_{5}+\beta_{5}\hat{P}_{jpt}+\gamma_{5}\ln\hat{R}_{ihjkpt}+\eta_{5}X_{ihjpt}+\phi_{5}M_{hjpt}+\varphi_{5}W_{jpt}+\delta_{j}+\theta_{t}+\mu_{pt}+\varepsilon_{ihjpt}$ \| (5) Here, $\hat{P}_{jpt}$ are predicted through equation (3) while $\ln\hat{R}_{ihjkpt}$ are predicted from equation (4).” “according to Levinson (2012), Ambrey et al. (2014) and Zhang et al. (2017a, 2017b), the WTP of affected residents on improved air quality (measured by reduced SO_2_ concentration) can be calculated by: $\hat{WTP}=\frac{\partial R}{\partial P}\left\vert\begin{matrix} \\ \\ dSWB=0 \end{matrix} \right.=-\bar{R}\frac{\hat{\beta}_{5}}{\hat{\gamma}_{5}}$ Here, $\bar{R}$ is the average income of the sample. Equation (3)~(7) are estimated using the CHIP data. Finally, with influence of TCZ policy on air pollution ($\hat{\beta}_{1}$) and monthly income of affected residents ($\hat{\beta}_{2}$) estimated in the first step and the residents’ WTP for less air pollution estimated in the second step, we can calculate the average net money value of TCZ policy on affected residents through: $Value\left( \Delta SWB \right)=\hat{\beta}_{1}\hat{WTP}+\hat{\beta}_{2}$” The related content can be found in page 22 - 27 of our replenished manuscript. Second, we adopted a series of robustness checks regarding the inconsistency in time and area between the two data sets. For time inconsistency: “Fourth, one concern is that the CHIP data are collected in a period different from the year of the policy shock, therefore the WTP estimated from CHIP data may be different from that of the policy shock year, if WTP changes over time. In this sense, we recalculate WTP by taking account of this concern. More specifically, according to equation (7), the WTP is determined by its two components: the ratio $\frac{\hat{\beta}_{5}}{\hat{\gamma}_{5}}$ and the average income $\bar{R}$. For the first component, we first regress equation (3) ~ (5) by year, to see how $\frac{\hat{\beta}_{5}}{\hat{\gamma}_{5}}$ changes overtime. Table 9 reports the by year results of equation (5).” “As shown in the table, the $\frac{\hat{\beta}_{5}}{\hat{\gamma}_{5}}$ ratio takes a special high value in the year 2002(1.04), then it dramatically decreased to a level around 0.20 in the year 2007, 2008, and 2013, and does not show a clear time trend then. There are two possibilities which might lead to such phenomena. First, it is possible that before 2002, the ratio is fluctuating around 1.04, and then a structural change in the correlation happens sometime between 2002 and 2007 which result in a new stable ratio around 0.2. In this case, we should use 1.04 to approximate the ratio in the policy shock year. Second, it is possible that the ratio is always around 0.2 and the 2002 result is an outlier. In this case, we should use the average ratio of the 2007-2013 estimates to approximate the ratio in the policy shock year. We try both. Together with a linear interpolation approach which predict the ratio in the policy shock year to be 0.322, we run 3 robustness checks for this concern. For the second component, we adopt the average income of subsamples from the affected areas in CHNS data. We report the related results in Table 10.” “As shown in the Table, our previous conclusion that the TCZ policy will reduce pollution at a cost of local income, but the environmental benefit dominates that income loss and thus improves SWB carries through in all three cases.” The related content can be found in page 36 - 38 of our replenished manuscript. For geographic inconsistency: “Finally, we account for the difference in geographic coverage between the two data sets we use. As mentioned in the data section, while the CHIP data covers all provinces of mainland China, the CHNS data only covers 10 provinces. In this sense, one might argue that the WTP estimated by CHIP data cannot represent the preference of the narrower CHNS provinces. To address this concern, we further use the propensity score method (PSM) to identify subsamples in the CHIP data which are similar to that of the CHNS provinces in terms of pollution levels, economic conditions, and weather conditions, the matching method adopted is the kernel matching with a bandwidth of 0.01. Table 11 presents the evaluation of matching effectiveness. As shown in the table, the p-values from the t-tests for pollution and GDP of the matched samples exceed 0.1, indicating no statistically significant differences between the matched city samples and the cities in CHNS.” “We then repeat what we did in Table 9 and 10 and report the related results in Table 12 and 13. Again, as shown in Table 12, the by year estimation gives an especially high $\frac{\hat{\beta}_{5}}{\hat{\gamma}_{5}}$ for the year 2002, while much lower ones in the following year. So, we adopted robust checks for 3 hypothetical scenarios: the linear interpolation scenario where we assume that $\frac{\hat{\beta}_{5}}{\hat{\gamma}_{5}}$ changed smoothly as time pass on at the average rate revealed in the CHIP data; the average scenario where we assume that the 2002 outcome is an outlier, and use the average $\frac{\hat{\beta}_{5}}{\hat{\gamma}_{5}}$ in the rest years to calculate WTP; and the structural change scenario, where we assume that $\frac{\hat{\beta}_{5}}{\hat{\gamma}_{5}}$ in the policy shock years are similar to that in the year 2002. As shown in Table 13, we again find that the environment improvement effect dominates the income reduction effect, and the TCZ policy brought a positive money value to the affected residents.” The related content can be found in page 38 - 40 of our replenished manuscript. |
| 2) Construction of the SWB function. In the model 1, it is clear that the SWB is predicted from the household health status, income, and a number of socio-economic characteristics. Since you also controlled for the same set of socio-economic characteristics in model (2), the predicted SWB equals to a composite indicator of health status and income. But in the follow-up analysis, you explain the effect of TCZ policy on SWB from the perspective of air pollution and income as in model (3) and (4). I wonder why don’t you just predict the SWB from the air pollution and income in model (1) to make it more consistent with the follow-up analysis? | We truly appreciate your advice that pollution and income could be incorporated into equation (1) to ensure consistency with the subsequent analysis.  We have combined this suggestion into our new empirical strategies:  First, we have changed our identification strategy and no longer trying to predict the SWB for CHNS interviewers using CHIP data. Instead, following the suggestion from the editor, we use a less complex approach to draw information from CHIP data and CHNS data. Details can be viewed in our response to your last concerns.  Second, in the second stage of the above-mentioned process, we use pollution instead of health to ensure consistency. Though we keep our results using health in the stage as a robustness check for mechanism. |
| 3) Construction of the fake data. I feel it odd to inflate the data by expanding 1 observation to 100, should this trick artificially creates some problems of autocorrelation? Why don’t you just use a simple linear probit model in the first step to predict the SWB for households with the original CHNS data? This is more clear and simple for implementation. | Thank you for highlighting the issues in our data processing methods. We agree with your concerns about potential autocorrelation problems caused by the 1:100 expansion method, which may artificially reduce standard errors and inflate significance levels. To address this, we have eliminated the use of expanded datasets and now rely solely on CHIP data for SWB-related analyses. Detailed methods can be viewed in our response to the previous two concerns.  Regarding your suggestion to use a simple linear probit model, we note that SWB is an ordered categorical variable with values of 1, 2, and 3. While linear probability models (LPMs) may simplify implementation, they often generate predicted probabilities outside the [0,1] range and are less suited for ordered outcomes. Therefore, we retained the use of ordered probit models for more accurate predictions. |
| 4) Identification of the TCZ effect. The key assumption of difference-in-difference approach is the parallel trend assumption. However, it is unclear how do you test this assumption from the main text, and the results in table 4 are doubtful without any figures to show the parallel trend. Moreover, it is also important to test the parallel trend assumption for the follow-up analysis of air pollution and income. These test results could be put into appendix. | Thank you for your valuable comments regarding the parallel trend assumption in the DID method. We fully acknowledge its importance. Since under our new identification strategy, we no longer regress SWB or predicted SWB directly on policy shocks, instead, we first analyze how policy shock affects pollution and income using DID approach and then evaluate how people make trade-offs between lower pollution and income reduction using Levison (2012)’s framework. Under the new empirical strategy, we only need to check the parallel trend for air pollution analysis and income analysis in equation (1) and (2).  We do as follow:  We first apply the traditional parallel trend test for equation (1), i.e., DID analysis regarding how policy shock pollution. We draw the related results in Figure 3.  “Figure 3 illustrates the results of parallel trend test for the DID analysis regarding the effect of TCZ policy on local SO_2_ concentration, i.e., equation (1). As shown in the figure, before policy shock, the difference between the treated group and the control group is not significant, but after shock, the difference becomes significant in most of the period. The figure supports the validity of our DID analysis.”  We then turn to parallel trend test for equation (2), i.e., DID analysis regarding how policy shock income. However, since income information is based on survey data and have a relatively lower frequency, traditional parallel trend test does not work well in this case.  “For the DID analysis regarding the effect of TCZ policy on income of affected residents, traditional parallel trend figure is not available, since the analysis is done using survey data which has limited number of periods to graph such figure. In this sense, we adopt the test framework of Bleakley (2007) and Wang et al. (2021) to test the parallel trend by setting a fake policy shock one period before the real shock (1998) to see if such fake shock (in 1997) has significant effects on the sub-sample before the real shock (1991-1997). The null hypothesis is the fake shock has significant effect, and reject it suggests the parallel trend. ”  “Table 5 reports the related results. As shown in Column (4) of the table, after controlling the appropriate variables and fixed effects, the effect of the fake shock becomes insignificant, which verifies the validity of our second DID analysis.”  The related content can be found in page 31 - 33 of our replenished manuscript. |
| 5) Misunderstanding in the heterogeneity analysis. How do you implement the heterogeneity analysis in section 5.2? Should you divide the full sample into subsamples or use interaction terms? It is unclear from reading the text, and in Table 5-7, it seems that you just simply add the classification variables in the model. You should clarify your approach. | Thank you for your comments on the heterogeneity analysis. We apologize for any confusion caused by the insufficient original description and have revised the text to clarify the methods and results of the heterogeneity analysis. Specifically, in Section 5.3, we take our heterogeneity analysis comparing the policy effects on developed cities vs. undeveloped cities as an example to introduce the related empirical strategies:  “For example, we first divide the whole sample cities into developed cities and undeveloped cities according to whether the GDP of the city is above sample average. We then generate a dummy variable which takes the value 1 if the city is a developed city and a value 0 otherwise. Next, we modify equation (1) (2) and (5) and regress:  $P_{jpt}=\alpha_{1}+\beta_{8}\left( {TCZ}_{j}\times{Post}_{t} \right)+\beta_{9}\left( {TCZ}_{j}\times{Post}_{t}\times{developed}_{j} \right)+\varphi_{1}W_{jpt}+\delta_{j}+\theta_{t}+\mu_{pt}+\varepsilon_{jt}$  $R_{ihcjpt}=\alpha_{2}+\beta_{10}\left( {TCZ}_{j}\times{Post}_{t} \right)+\beta_{11}\left( {TCZ}_{j}\times{Post}_{t}\times{developed}_{j} \right)+\eta_{2}X_{ihcjpt}+\phi_{2}M_{chjpt}+\nu_{2}D_{cjpt}+\varphi_{2}W_{jpt}+\delta_{j}+\theta_{t}+\mu_{pt}+\varepsilon_{ihcjpt}$  and:  ${SWB}_{ihjkpt}=\alpha_{5}+\beta_{12}\hat{P}_{jpt}+\beta_{13}\left( \hat{P}_{jpt}\times{developed}_{j} \right)+\gamma_{8}\ln\hat{R}_{ihjkpt}+\gamma_{9}(\ln\hat{R}_{ihjkpt}\times{developed}_{j})+\eta_{5}X_{ihjpt}+\phi_{5}M_{hjpt}+\varphi_{5}W_{jpt}+\delta_{j}+\theta_{t}+\mu_{pt}+\varepsilon_{ihjpt}$”  The effects of the policy on undeveloped city is given by $\hat{\beta}_{8}$ and $\hat{\beta}_{10}$ while the effect of the policy on developed cities is given by $\hat{\beta}_{8}+\hat{\beta}_{9}$ and $\hat{\beta}_{10}+\hat{\beta}_{11}$. The WTP for undeveloped cities is given by $\hat{WTP}_{undeveloped}=-\bar{R}_{undeveloped}\frac{\hat{\beta}_{12}}{\hat{\gamma}_{8}}$, while the WTP for developed cities is given by $\hat{WTP}_{developed}-\bar{R}_{developed}\frac{\hat{\beta}_{12}+\hat{\beta}_{13}}{\hat{\gamma}_{8}+\hat{\gamma}_{9}}$. The money value the TCZ policy brought to residents of the two types of cities are $\hat{\beta}_{8}\hat{WTP}_{undeveloped}+\hat{\beta}_{10}$ and $(\hat{\beta}_{8}+\hat{\beta}_{9})\hat{WTP}_{developed}+(\hat{\beta}_{10}+\hat{\beta}_{11})$ respectively.”  The related content can be found in page 40 - 41 of our replenished manuscript. |
| 6) The endogeneity problem. You mention that two instruments are employed to address the endogeneity issue of air pollution in Equation (8). I guess that you mean the Equation (5). If you think that the air pollution here is endogenous, it is also the concern for income. You should address the endogeneity problems of both air pollution and income with additional IVs. You should also carefully discuss the relevance and exclusive restriction of your IVs and present the first stage results of the IV estimations, which is now absent in the text. | Thank you for your valuable comments on the endogeneity problem in our research. We fully recognize the issue and applied IV method for both air pollution and income. “Since both air quality and income might be correlated with factors which also influence SWB, we apply a series of instrumental variables to deal with potential endogeneity issues.” “For air quality, which is measured by the pollution concentration level, we use the number of days in the year when the second layer of the atmosphere is warmer than the first layer and the number of days when the third layer of the atmosphere is warmer than the first layer as instrumental variables. Such IVs are widely adopted in studies evaluating economic consequences of air pollution (see Jans et al., 2018; Fu et al., 2021; Wang et al., 2021 and Chen et al., 2022). In terms of individual income, we choose the provincial level average wage of workers who work in the same industry as the instrument variable for individual income, as suggested by Zhang et al. (2017 a). We use a 2SLS approach to estimate this IV model. The 1^st^ stage regressions are: \| $P_{jpt}=\alpha_{3}+\beta_{3}{Days}_{jpt}+\gamma_{3}{Wind}_{jpt}+\varphi_{3}W_{jpt}+\delta_{j}+\theta_{t}+\mu_{pt}+\varepsilon_{jt}$ \| (3) and: \| $lnR_{ihjkpt}=\alpha_{4}+\beta_{4}ln{Wage}_{kpt}+\eta_{4}X_{ihjt}+\phi_{4}M_{hjpt}+\varphi_{4}W_{jpt}+\delta_{j}+\theta_{t}+\mu_{pt}+\varepsilon_{ihjpt}$ \| (4) In equation (3), ${Days}_{jpt}$ is the weighted average of number of days in the year when the second layer of the atmosphere is warmer than the first layer and that when the third layer of the atmosphere is warmer than the first layer. The weight is 1:1. ${Wind}_{jpt}$ is the annual average wind speed of the city. In equation (4), $lnR_{ihjkpt}$ is the log income of individual $i$ at time $t$, where individual $i$ is from household $h$, lives in the community $c$ of city $j$ in province $p$, and works for industry $k$. $ln{Wage}_{kpt}$ is the average log wage for staffs who work in the same industry and the same province as individual $i$. Other control variables are the same as in equation (1) and (2). In the 2^nd^ stage, we regress the following ordered choice model: \| ${SWB}_{ihjkpt}=\alpha_{5}+\beta_{5}\hat{P}_{jpt}+\gamma_{5}\ln\hat{R}_{ihjkpt}+\eta_{5}X_{ihjpt}+\phi_{5}M_{hjpt}+\varphi_{5}W_{jpt}+\delta_{j}+\theta_{t}+\mu_{pt}+\varepsilon_{ihjpt}$ \| (5) Here, $\hat{P}_{jpt}$ are predicted through equation (3) while $\ln\hat{R}_{ihjkpt}$ are predicted from equation (4).” For both scenarios, we reported the 1^st^ stage results in Table 4 to verify that the IVs chosen are relevant. The exclusiveness of the IVs is supported by existing literature. The related content can be found in page 24 - 26 of our replenished manuscript. Thank you again for your guidance, which has greatly improved the robustness of our research. |
| 7) Contribution of the paper. In the literature, there are a number of papers to estimate the impact of TCZ policy in China. Why your paper is novel compared to other TCZ studies? You should strengthen your contribution to the literature in the introduction. | Thank you for your thoughtful comments on the contribution of our paper. We have strengthen our statement of contribution, especially regarding the difference between our paper and others that also evaluate the effects of TCZ policies.  “Abundant research also studied the effect of TCZ policy. However, due to data limitations, most of the existing literature have to choose focusing on a single aspect of the policy effects. For example, Zheng et al. (2005), Huang et al. (2022), Wang et al. (2023), and Yang et al. (2023) all focus on the pollution reduction or health effect of the policy, while Sun et al. (2019) and Li et al (2024) focus on the policy shock to income.  Our research extends the environmental regulation literature and the relevant literature on the TCZ policy by: 1) comprehensively examining the two mechanisms (pollution/health effect and income effect) through which the TCZ policy affects SWB in the context of the Easterlin paradox and 2) calculating the monetary value the TCZ policy brought to affected residents using a WTP-based approach.”  The related content can be found in page 12 of our replenished manuscript. |

Response of Authors to the Comments of Reviewer #2

“Wealth, Health, and Happiness: An Inverse Story of the Easterlin Paradox in China”

| **Reviewer’s Comments** | **Authors’ Response** |
| --- | --- |
| Comments to the Author  MAJORL COMMENTS  1) The biggest concern is that the authors did not report the details of the estimation results to generate fake dataset, although the authors provided a clear methodological process. Authors graphically demonstrated the marginal effects of income and self-reported health status on the probabilities to choose three levels of SWB in the CHIPS data but omitted other details, noting “instead of reporting the meaningless estimates on coefficients (p31).” The problem is not either the coefficients or marginal effects are better. It is that all other information is not disclosed. More information is needed, such as the significances and signs of other variables (or ME), goodness of fit and/or overall explanation power, sample size and sample-selection criteria, etc. Although the overall results of this study were interesting, it was hard to agree or disagree with them if the derivation of the fake dataset is mostly kept in a blackbox.  Further concerns are on their 1:100 expansion method. The authors noted “that this trick is just to deal with the problems caused by the particular characteristic of ordered choice model, otherwise, what we do is nothing different from predicting a missing SWB and applying it for further research, which is common in researches facing incomplete data problems (see Little and Ann 2004, Kang and Schafer 2007, Penn 2009).” However, it is not that simple. | We sincerely appreciate your detailed and in-depth comments. After carefully considering opinions from the editor and two reviewers, we have abandoned the 1:100 expansion approach and adopted a less complex and less controversial approach as suggested by the editor to identify the combined effect of TCZ policy on SWB. The main process are as follows: “First, we use a typical difference in difference (DID) approach to estimate the effect of the TCZ policy on air pollution and residential income of affected cities. For air pollution, we regress: \| $P_{jpt}=\alpha_{1}+\beta_{1}\left( {TCZ}_{j}\times{Post}_{t} \right)+\varphi_{1}W_{jpt}+\delta_{j}+\theta_{t}+\mu_{pt}+\varepsilon_{jt}$ \| (1) Here, $P_{jpt}$ is the air pollution of city $j$ (which locates in province $p$) at time $t$, measured by average ambient concentration of SO_2_ at the city level. ${TCZ}_{j}$ is a dummy variable indicating whether the observation is from a city covered by the TCZ policy (takes the value of 1 if it is) while ${Post}_{t}$ is a dummy variable indicating whether the observation is from the post-policy period (takes the value of 1 in the year 1998 and so after). $\hat{\beta}_{1}$ is then the parameter of interest which illustrates the effect of TCZ policy on city air pollution. In this regression, we control a series of city level meteorological factors and economic factors such as temperature, precipitation and logarithm of local GDP, the city fixed effect $\delta_{j}$, the time fixed effect $\theta_{t}$, and the combined fixed effect at a higher dimension, i.e., $\mu_{pt}={Province}_{j}\times{time}_{t}$. $\varepsilon_{jpt}$ is a heteroskedasticity-robust standard error term. For residential income, we regress: \| $R_{ihcjpt}=\alpha_{2}+\beta_{2}\left( {TCZ}_{j}\times{Post}_{t} \right)+\eta_{2}X_{ihcjpt}+\phi_{2}M_{chjpt}+\nu_{2}D_{cjpt}+\varphi_{2}W_{jpt}+\delta_{j}+\theta_{t}+\mu_{pt}+\varepsilon_{ihcjpt}$ \| (2) Here, $R_{ihcjpt}$ is the average monthly income of individual $i$ at time $t$, where the individual is from household $h$ and lives in the community $c$ of city $j$ in province $p$. $X_{ihcjpt}$ is a series of individual level control variables including the education year, gender, ethnicity and age. $M_{chjpt}$ is a series of household level control variables including family size and the household’s income. $D_{cjpt}$ is a series of community level control variables including the population density and the market composition index. $W_{jpt}$, $\delta_{j}$, $\theta_{t}$ and $\mu_{pt}$ are the same as in equation (1), and $\hat{\beta}_{2}$ is the parameter of interest which indicates the effect of TCZ policy on monthly income of affected individuals. The above two regressions are estimated using the CHNS data. Second, following the wisdom of Levinson (2012), Ambrey et al. (2014) and Zhang et al. (2017a, 2017b), we estimate residents’ WTP for improved air quality by exploring their trade-offs between economic benefit and environmental quality subject to the constraint that their SWB stay the same. Since both air quality and income might be correlated with factors which also influence SWB, we apply a series of instrumental variables to deal with potential endogeneity issues. We also use the methods adopted by the ordered choice models to take care of the potential problems from the discrete choice issues following Dolan et al. (2008), Diener et al. (2018) and Clark (2018).” “We use a 2SLS approach to estimate this IV model. The 1^st^ stage regressions are: \| $P_{jpt}=\alpha_{3}+\beta_{3}{Days}_{jpt}+\gamma_{3}{Wind}_{jpt}+\varphi_{3}W_{jpt}+\delta_{j}+\theta_{t}+\mu_{pt}+\varepsilon_{jt}$ \| (3) and: \| $lnR_{ihjkpt}=\alpha_{4}+\beta_{4}ln{Wage}_{kpt}+\eta_{4}X_{ihjt}+\phi_{4}M_{hjpt}+\varphi_{4}W_{jpt}+\delta_{j}+\theta_{t}+\mu_{pt}+\varepsilon_{ihjpt}$ \| (4) In equation (3), ${Days}_{jpt}$ is the weighted average of number of days in the year when the second layer of the atmosphere is warmer than the first layer and that when the third layer of the atmosphere is warmer than the first layer. The weight is 1:1. ${Wind}_{jpt}$ is the annual average wind speed of the city. In equation (4), $lnR_{ihjkpt}$ is the log income of individual $i$ at time $t$, where individual $i$ is from household $h$, lives in the community $c$ of city $j$ in province $p$, and works for industry $k$. $ln{Wage}_{kpt}$ is the average log wage for staffs who work in the same industry and the same province as individual $i$. Other control variables are the same as in equation (1) and (2). In the 2^nd^ stage, we regress the following ordered choice model: \| ${SWB}_{ihjkpt}=\alpha_{5}+\beta_{5}\hat{P}_{jpt}+\gamma_{5}\ln\hat{R}_{ihjkpt}+\eta_{5}X_{ihjpt}+\phi_{5}M_{hjpt}+\varphi_{5}W_{jpt}+\delta_{j}+\theta_{t}+\mu_{pt}+\varepsilon_{ihjpt}$ \| (5) Here, $\hat{P}_{jpt}$ are predicted through equation (3) while $\ln\hat{R}_{ihjkpt}$ are predicted from equation (4).” “according to Levinson (2012), Ambrey et al. (2014) and Zhang et al. (2017a, 2017b), the WTP of affected residents on improved air quality (measured by reduced SO_2_ concentration) can be calculated by: $\hat{WTP}=\frac{\partial R}{\partial P}\left\vert\begin{matrix} \\ \\ dSWB=0 \end{matrix} \right.=-\bar{R}\frac{\hat{\beta}_{5}}{\hat{\gamma}_{5}}$ Here, $\bar{R}$ is the average income of the sample. Equation (3)~(7) are estimated using the CHIP data. Finally, with influence of TCZ policy on air pollution ($\hat{\beta}_{1}$) and monthly income of affected residents ($\hat{\beta}_{2}$) estimated in the first step and the residents’ WTP for less air pollution estimated in the second step, we can calculate the average net money value of TCZ policy on affected residents through: $Value\left( \Delta SWB \right)=\hat{\beta}_{1}\hat{WTP}+\hat{\beta}_{2}$” The related content can be found in page 22 - 27 of our replenished manuscript. Also, as you suggested, while reporting related results, we have included all the mentioned information. |
| 2) It does not seem that the authors dealt with the decreased standard errors owing to the increased sample size by 100-time appearance of each individual. That procedure would lower the standard errors and the t-values of the variables would be inflated. The authors did not state anything about this issue, and did not cite any study that utilize similar 1:100 expansion. I suspect that, after appropriate treatment of the standard errors, some of the coefficients that have values close to zero will become insignificant. | We sincerely appreciate your pointing out the problems in our research, which has extremely important guiding significance for us to improve our research. In view of this, we have made significant adjustments in the revised research. In particular, we no longer use the CHIP data to predict the SWB for CHNS interviewees, and there is no need to increase the sample size by 100-time appearance of each individual.  The detailed approach of the new empirical strategy can be viewed in our response to your last concern. |
| 3) Further, why did the authors use non-linear model in the first stage but linear model in the second-stage estimations, although the dependent variable is the same SWB? Clearly, the SWB is a categorical measure. But in the second stage, the authors anyway mainly used “traditional fixed effect DID” and treated the SWB as a continuous variable. 1) If the non-linear model is preferable, then the second stage should also use non-linear model. 2) But if a linear-model is sufficient, then the authors can use linear model in the first stage as well and do not need to apply 1:100 expansion. What if the authors use a linear regression in the first stage, predict SWB^hat of CHNS individuals (which will be mostly non-integer values), and regress SWB^hat in the second stage without 1:100 expansion. | We sincerely appreciate your profound and detailed comments, which play a crucial role in improving our research.  The reason why in the second stage we use a “traditional fixed effect DID” is because in that stage, we do not need details about how changes in income and pollution affect individuals’ probability of being happy, fair or not happy, instead, we focus on individual’s tradeoff between income and pollution to hold SWB constant.  Based on your comments and after careful consideration, we have made significant adjustments in the revised research, and we no longer use the CHIP data to predict the SWB for CHNS interviewees. Now, in our 3-stage approach mentioned previously, we just need to estimate individual’s tradeoff between income and pollution holding SWB constant, so we adopt a traditional linear framework there as suggested by Levinson (2012), Ambrey et al. (2014) and Zhang et al. (2017a, 2017b).  More details can be viewed in our response to your concern (1). |
| 4) The authors cited three papers, but they basically focus on cases where key variables are missing for subsamples. But in this study, the key variable, SWB, is completely missing in the CHNS data and predicted from CHIP data. I felt that “nothing different” is not reasonable. For example, an underlying assumption to extrapolate the CHIP-based equation to the CHNS data is that the preferences of people in the CHIPS (starting from 2003) and the CHNS (1991–2006) are unchanged. But is it justifiable? In particular, a marginal effect of a one-yuan increase in income on the SWB could be different over time. | Thank you for your valuable comment. As mentioned previously, we have abandoned the complex approach trying to predict the SWB for CHNS interviewees using the CHIP data. The problem of this concern is greatly mitigated. More details regarding our new identification strategy can be viewed in our response to your concern (1).  Of course, even in our new empirical strategy, we still need to use CHNS data to estimate how the TCZ policy affect income and pollution while using CHIP data to estimate how affected resident’s tradeoff between income and pollution. To address the concern that the two data sets are not from the same time periods and people’s preference may change over time we made the following robustness checks.  “Fourth, one concern is that the CHIP data are collected in a period different from the year of the policy shock, therefore the WTP estimated from CHIP data may be different from that of the policy shock year, if WTP changes over time. In this sense, we recalculate WTP by taking account of this concern. More specifically, according to equation (7), the WTP is determined by its two components: the ratio $\frac{\hat{\beta}_{5}}{\hat{\gamma}_{5}}$ and the average income $\bar{R}$. For the first component, we first regress equation (3) ~ (5) by year, to see how $\frac{\hat{\beta}_{5}}{\hat{\gamma}_{5}}$ changes overtime. Table 9 reports the by year results of equation (5).”  “As shown in the table, the $\frac{\hat{\beta}_{5}}{\hat{\gamma}_{5}}$ ratio takes a special high value in the year 2002(1.04), then it dramatically decreased to a level around 0.20 in the year 2007, 2008, and 2013, and does not show a clear time trend then. There are two possibilities which might lead to such phenomena. First, it is possible that before 2002, the ratio is fluctuating around 1.04, and then a structural change in the correlation happens sometime between 2002 and 2007 which result in a new stable ratio around 0.2. In this case, we should use 1.04 to approximate the ratio in the policy shock year. Second, it is possible that the ratio is always around 0.2 and the 2002 result is an outlier. In this case, we should use the average ratio of the 2007-2013 estimates to approximate the ratio in the policy shock year. We try both. Together with a linear interpolation approach which predict the ratio in the policy shock year to be 0.322, we run 3 robustness checks for this concern. For the second component, we adopt the average income of subsamples from the affected areas in CHNS data. We report the related results in Table 10.”  “As shown in the Table, our previous conclusion that the TCZ policy will reduce pollution at a cost of local income, but the environmental benefit dominates that income loss and thus improves SWB carries through in all three cases.  “Finally, we account for the difference in geographic coverage between the two data sets we use. As mentioned in the data section, while the CHIP data covers all provinces of mainland China, the CHNS data only covers 10 provinces. In this sense, one might argue that the WTP estimated by CHIP data cannot represent the preference of the narrower CHNS provinces. To address this concern, we further use the propensity score method (PSM) to identify subsamples in the CHIP data which are similar to that of the CHNS provinces in terms of pollution levels, economic conditions, and weather conditions, the matching method adopted is the kernel matching with a bandwidth of 0.01. Table 11 presents the evaluation of matching effectiveness. As shown in the table, the p-values from the t-tests for pollution and GDP of the matched samples exceed 0.1, indicating no statistically significant differences between the matched city samples and the cities in CHNS.”  “We then repeat what we did in Table 9 and 10 and report the related results in Table 12 and 13. Again, as shown in Table 12, the by year estimation gives an especially high $\frac{\hat{\beta}_{5}}{\hat{\gamma}_{5}}$ for the year 2002, while much lower ones in the following year. So, we adopted robust checks for 3 hypothetical scenarios: the linear interpolation scenario where we assume that $\frac{\hat{\beta}_{5}}{\hat{\gamma}_{5}}$ changed smoothly as time pass on at the average rate revealed in the CHIP data; the average scenario where we assume that the 2002 outcome is an outlier, and use the average $\frac{\hat{\beta}_{5}}{\hat{\gamma}_{5}}$ in the rest years to calculate WTP; and the structural change scenario, where we assume that $\frac{\hat{\beta}_{5}}{\hat{\gamma}_{5}}$ in the policy shock years are similar to that in the year 2002. As shown in Table 13, we again find that the environment improvement effect dominates the income reduction effect, and the TCZ policy brought a positive money value to the affected residents.”  The related content can be found in page 36 - 40 of our replenished manuscript. |
| 5) The main results and various robustness check and heterogeneity analyses showed that the TCZ policy improved the SWB. Then the authors argued that the positive effect is “evidence supporting the environmental explanation of the Easterlin paradox from an inverse story logistic (p32).” But the positive coefficient itself does not explain anything about the Easterlin Paradox, because at this moment, the authors have not provided any evidence that the TCZ policy simultaneously improved environmental quality and lowered economic welfare. The authors argue so conceptually in Figure 3 (p21), but the summary statistics (p25) rather suggest the opposite: the TCZ policy improves both the environmental quality and wage rates. It is Table 8 (pp43¬-44) where the authors provided evidence that the TCZ policy simultaneously improved environmental quality and lowered economic welfare for the first time (although several questions exist for this result, see below). Therefore, under the current structure, it was hard to agree with the abovementioned claim in p32. In the conclusion section, the authors explain the results of Table 8 first and then the impact of the TCZ policy on the SWB. The results section should proceed in the same way.  Section 5.3 is directly related to the inverse story of the Easterlin Paradox. While the WTP calculation method itself is fine, the estimation is questionable. | We sincerely appreciate your valuable comments on the structure and argumentation logic of our paper. According to your suggestion, we made a series of adjustments to better express our idea and to avoid causing confusions.  First, in summary statistics, we no longer make comparisons on primary statistics across treated and control groups, the results of which is neither accurate nor helpful in supporting our hypothesis.  Second, we adjusted the structure of our results section to better incorporate with the theoretical framework of Easterlin paradox. While presenting our main results, we proceed as you suggested.  “We first apply the DID approach to the CHNS data to show that the implementation of the TCZ policy has significantly improved the air quality in affected cities, though at a cost of local residents’ income. Table 2 reports our results regarding the effect of the TCZ policy on air quality measured by the city level SO_2_ concentration.”  “As shown in Column (3) of the table, after controlling the time fixed effect, the city fixed effect, and the province by year fixed effect, we find a negative correlation between the implementation of the TCZ policy and the city level SO_2_ concentration which is significant at 1% level. More specifically, the implementation of the policy caused the SO_2_ concentration in the affected cities to decrease by about 0.89 μg/m^3^.”  “Table 3 illustrates our results regarding the economic influence of the TCZ policy on affected cities measured by the average monthly income of surveyed individuals. As shown in Column (4) of the table, after controlling factors that are possible to affect individual income, the TCZ policy is found to have a negative effect on the monthly income of affected residents. More specifically, the implementation of the TCZ policy reduced the monthly income of affected residents by about ￥26, this estimate is significant at 5% level.”  “We then estimate the CHIP data to explore the relationship between income, air quality and SWB using a 2SLS IV regression. We report the related results in Table 4, where Column (1) and (2) represent the 1^st^ stage results while Column (3) represents the 2^nd^ stage results. As shown in the table: (1) in Column (1) and (2), the two IV we choose both have significant effects on endogenous variables, and the F statistics for weak IV test reject the null hypothesis that the IVs we choose are weak IVs; (2) in Column (3), we get $\hat{\beta}_{5}=-0.058$ and $\hat{\gamma}_{5}=0.241$. Since we use OLS model in this regression, these two estimate do not have direct economic meanings, yet their proportion reveals that to keep the probability the individual feels excellent, good, fair, poor, or very poor the same what trade-off should be made between income and pollution. Together with the average income of the sample, i.e., ￥778.2 per month, we estimate the average monthly willingness to pay for per μg/m^3^ reduction of SO_2_ concentration be￥187.2.”  “Finally, we combine our DID results and IV results to estimate the money value of the net average SWB improvements the TCZ policy yields annually for affected residents…”  The related content can be found in page 27 - 30 of our replenished manuscript. |
| 6) In column (26), the sample size is 1245170, meaning that the fake dataset was used. But in this estimation, because the SWB is not used, the authors should directly use the original CHNS dataset and do not have to use the fake dataset in which the same individuals appear 100 times. I wonder if the TCZxPost remains significant if the original CHNS dataset is used. Indeed, the summary statistics showed that the wage rate grew faster in the TCZ cities than in the non-TCZ cities, suggesting that the TCZ policy increased wage rate and lowered the pollution level. Maybe the effect of TCZ policy was reverted in column (26) after controlling for other factors (then it supports the inverse story of Easterlin Paradox). But the significant coefficient may just reflect the small standard errors caused by the 1:100 expansion. In sum, because there is no need to use the fake dataset, the authors should try this estimation with the original CHNS dataset. | We sincerely appreciate your detailed and in-depth comments.  Under our new identification strategies (details of which can be found in our response to your concern (1)), we directly use the original CHNS dataset to explore how the TCZ policy affect income and air pollution with equation (1) and (2). And the related results are reported in Table 2 and 3.  As shown in Table 3, after controlling for a series of individual, family community and city level characteristics, weather factors, the city fixed effects, the year fixed effects and the province -by-year fixed effects, the TCZ policy brought a significantly negative effect on individual income, which is consistent with the Easterlin Paradox.  The related content can be found in page 28 - 29 of our replenished manuscript. |
| 7) In column (27), the SWB is regressed to SO2 level and log(wage) based on the fake dataset. But because the TCZ policy variable is not used this time, the authors can use the original CHIP dataset for this analysis. Do these coefficients remain significant if the original CHIPS data are used and the sample size is not artificially increased by 100 times? | We sincerely appreciate your valuable comments.  Under our new identification strategies (details of which can be found in our response to your concern (1)), we used the original CHIP data to estimate how people tradeoff between income and air quality. The related results are reported in column (3) of Table 4.  As shown in the table, the effect of income on SWB is still significant at 1% level, while the effect of SO_2_ on SWB remains significant at 10% level. Directions of both effects are consistent with theoretical expectations.  The related content can be found in page 29 - 30 of our replenished manuscript. |
| 8) Further, the estimation of column (27) uses an IV. The IV itself is fine (used widely in the literature), I wonder why endogeneity matters. The dependent variable this time, SWB^hat, is basically predicted from the observable characteristics and unobservable factors cannot influence SWB^hat. Even if the authors continue using an IV, further information is needed, such as first-stage results, F-value, etc. | We sincerely appreciate your questions and suggestions regarding the use of instrumental variables in our research, which are extremely crucial for us to further optimize the research methods and presentation.  Under our new identification strategies (details of which can be found in our response to your concern (1)), we used the original CHIP data to estimate how people tradeoff between income and air quality. Since this time real data instead of the fake data are used. We continue to use IV in this scenario.  According to your suggestions, related information including the first-stage results and the related F-values are all included in our Main Results section (Section 5.1)  The related content can be found in page 29 - 30 of our replenished manuscript. |
| 9) The authors used the 1:100 expansion, so the sample size should be multiples of 100. But I saw the sample size of 1245170, 1246489, etc. Why? Is the CHIPs data also used as samples without multiplication? | We sincerely appreciate your questions about the details of our sample data. In the process of our previous 1:100 sample expansion, due to the rounding of the probabilities of different values of SWB, the number of expansions for some individuals was not exactly 100 times. Specifically, some individuals were expanded to 99 times, while others were expanded to 101 times, resulting in the final sample size showing numbers like 1245170 and 1246489 that are not multiples of 100 as you saw.  But under our new identification strategies (details of which can be found in our response to your concern (1)), data expansion is no longer used, therefore, this should not be a problem anymore. |
| OTHER COMMENTS  1) The literature review and background sections were too lengthy, spanning from p7–p21. The authors should shorten these parts by reducing irrelevant information. Further, in these sections, citation is incorrect or simply missing in the reference list. For example, DiMaria and Sarracino (2019), Naghdi et al. (2014), and Stelzner (2021) are the in-text citation (pp7–8), but according to the reference list, they should be DiMaria and Sarracino (2020), Naghdi et al. (2021), and Stelzner (2022). In p15, World Bank (2015), NBS (1991; 2007), and Ministry of Ecology and Environment, PRC, 1996) are cited, but they do not appear in the reference list. These are just examples, and there are other papers inaccurately cited. Careful re-checking is needed. | We sincerely appreciate your questions and suggestions regarding the length of the introduction and the citation of references. For the background sections, we have streamlined it, reducing the page length from 8 pages to 4 pages. Regarding the incorrect citations you mentioned, we have made corrections. Specifically, we changed "DiMaria and Sarracino., 2019" on line 18 of page 7 to "DiMaria and Sarracino., 2020"; changed "(Naghdi et al., 2014)" on line 7 of page 8 to "(Naghdi et al., 2021)"; changed "(Stelzner, 2021)" on line 9 of page 8 to "(Stelzner, 2022)". We have carefully checked the citations in the article and found and corrected other incorrect places. For example, we changed "(Zhang et al., 2017; Bakkeli, 2019)" on line 14 of page 5 to "(Zhang et al., 2017a; Bakkeli, 2020)"; changed "(Wolbring et al., 2011; Melzer et al., 2017)" on line 15 to "(Wolbring et al., 2013; Melzer and Muffels, 2017)"; changed "Wiese, 2017" on line 17 to "Wiese, 2014"; changed " Distante, 2012" on line 18 to "Distante, 2013"; changed " FitzRoy and Nolan, 2021" on line 18 to " FitzRoy and Nolan, 2022"; changed "(Brereton et al., 2007)" on line 12 of page 8 to "(Brereton et al., 2008)"; changed "Veenhoven and Vergunst (2013)" on line 9 of page 9 to "Veenhoven and Vergunst (2014)"; changed " (Sarracino et al., 2021)" on line 2 of page 10 to " (Sarracino et al., 2022)"; changed "Zhang et al. (2017)" on line 19 of page 10 to "Zhang et al. (2017a)"; changed "(DeVries et al., 2016)" on line 6 of page 21 to "(DeVries et al., 2017); and changed "Perneger and Bovier, 2004" on line 11 of page 21 to " Perneger et al., 2004".  Regarding the problem that the in-text citations do not have corresponding entries in the reference list, we have made the following changes:  (1) Due to the simplification of the background section, the citations of World Bank (2015), NBS (1991; 2007) have been removed.  (2) Changed "(NBS, 1996)" on line 13 of page 17 to "(CEYEC, 1996)"; and added the following to the reference section: "China Environment Yearbook Editorial Committee. (1996). China Environment Yearbook. China Environment Yearbook Press". The reason is that the national SO2 emission data involved in the article comes from the corresponding year's China Environment Yearbook. For example, the emission data for 1990 comes from the China Environment Yearbook published in 1991, which is mainly sponsored by the Ministry of Ecology and Environment of the People's Republic of China and edited and published by the China Environment Yearbook Press, not the National Bureau of Statistics.  (3) Changed "(Ministry of Ecology and Environment, PRC, 1996)" on line 5 of page 18 to "(Liu et al., 1998)" and added the following to the literature section: "Liu, B. J., Hao, J. M., He, K. B., et al. (1998). Research on the zoning and implementation policies of acid rain and sulfur dioxide pollution control areas in China. China Environmental Science, 18(1), 2 - 8." |
| 2) In pp 19–21 where the theoretical channels are discussed, the authors explain the possibilities that environmental regulation can positively affect income, not only the possibility that the regulation hinders income. It is nice. But I think there is an additional channel: an improved health and environment quality increase the income by improving labor productivity and labor supply amount. There are a lot of studies examining this point, such as  Aragón, F.M., Miranda, J.J., Oliva, P., 2017. Journal of Environmental Economics and Management 86, 295–309.  Borgschulte, M., Molitor, D., Zou, E.Y., (forthcoming). The Review of Economics and Statistics.  Fu, S., Viard, V.B., Zhang, P., 2022.  Although this channel is not the main one this study considers, a brief mentioning to this channel may improve the clarity of the conceptual flow. | We sincerely appreciate your detailed and highly constructive review comments. We fully agree with your view and have made corresponding modifications as follows:  (1) In the main text: Added on line 13 of page 21: “Improved health and environment quality are also found to be helpful in increasing labor productivity, total labor hours a worker can supply and thus the total wage residents can earn (Aragón et al., 2017; Borgschulte et al., 2024; Fu et al., 2022)”  (2) In the reference section: Added the following:  Aragón, F.M., Miranda, J.J., Oliva, P. (2017). Particulate matter and labor supply: The role of caregiving and non-linearities. Journal of Environmental Economics and Management, 86, 295 - 309.  Borgschulte, M., Molitor, D., & Zou, E. (2024). Air Pollution and the Labor Market: Evidence from Wildfire Smoke. The Review of Economics and Statistics, 106(6), 1558 - 1575.  Fu, S., Viard, V. B., & Zhang, P. (2022). Trans - boundary air pollution spillovers: Physical transport and economic costs by distance. Journal of Development Economics, 155, 102808. |
| 3) Estimation equations and variable notations need revision. Equation (1) is a linear equation and is not an ordered-choice equation. Equation (2) is the main second-stage equation, but it needs the term for fixed effects if fixed effects model is actually used in the results. | We sincerely appreciate your careful review and valuable comments. In the revised article, we have updated our equations according to your suggestions. In particular, we use $\delta_{j}$ to denote city fixed effects, $\theta_{t}$ to denote time fixed effects, and$\mu_{pt}$to represent province - year fixed effects, and explain the meanings and functions of these fixed effects in detail. |
| 4) In p 27, the authors wrote “We use the ordered logit model to estimate Equation (1), and the result tells us, conditional on income, health and other individual, household and city characteristics, what’s the probability an individual will say good, fair, or poor when asked: “Do you feel happy now?”. To make the two data sets consistent, we convert the four-level rank in CHIP data to three levels: excellent is converted into good, good is converted into fair, and poor and very poor are converted into poor.” But what variable are you talking about? Context-wise, it sounds like the SWB level was converted, but this cannot be true because CHNS data do not have SWB and consistency does not matter? Are you talking about household income? | We are very sorry for the parts of our paper that are not expressed clearly and precisely. Regarding the data conversion issue mentioned on page 27 of the original paper, since the self-reported health in CHNS has three levels, we actually performed the conversion operation on the SWB of CHIP in order to maintain a certain degree of logical coherence and comparability between the SWB of CHIP and the self-reported health of CHNS. In the revised manuscript, since we no longer predict SWB based on self-reported health, we have deleted the parts with unclear expressions. |
| 5) PSM is used in column (2), but more information is needed, such as the equation to estimate the PSM, matching method (nearest neighbor? Kernel? Or other?). | We sincerely appreciate your comments and requirements regarding the application of PSM in our research. In the original research, in the PSM matching process, the variables used were consistent with those in model 2. This ensured the coherence and comparability of variable selection under different analysis methods, making the research results more stable and persuasive. Regarding the matching method, we used the nearest neighbor matching. The nearest neighbor matching has the characteristics of being intuitive and effective in dealing with sample matching problems and can provide suitable control samples for our research to further explore the causal relationship between relevant variables. |
| 6) In Table 7, cities with high proportion of primary industry are labelled as “agricultural.” But it is counterintuitive that the TCZ policy worsened the SWB of people. But I think primary industry also includes mining. So it should be labelled as “agricultural and mining”. And it is very intuitive that the TCZ policy worsened the SWB of people in mining sector. | We sincerely appreciate your valuable comments on the classification and labeling of cities in Table 7 in our original research. To avoid confusion, this part of heterogeneity analysis has been removed from our discussions. |
| 7) In p43, “In estimating Equation (8), we use […] we report the results in Column (27)”. But in equation (8), there is nothing to additionally estimate: beta^hat3 and beta^hat4 are estimated based on equations (3) and (4), and WTP^hat is calculated from equations (5)–(7). Perhaps the author examined an additional equation (not equation 8) and showed the results in column (27). Careful revision of the explanation is needed. | We sincerely appreciate your pointing out the inconsistent problem between the equation annotation and the explanation in the text. In the original text, when it was mentioned that “when estimating equation (8) … and reporting the results in column (27)”, it should actually be equation (5). The citation of equation (8) here was a wrong reference, and equation (5) is the correct equation related to the subsequent operations and the results in column (27).  We deeply apologize for this oversight and have carefully corrected all the places with this wrong reference in the paper revision process to ensure the accuracy and consistency of the equation annotation, estimation, and result presentation, making the logic of the article clearer and more coherent. |
| 8) Further, the notation of ΔWTP^hat is a bit problematic. WTP^hat can take only the values of 1,2,3. So ΔWTP^hat being 649.499 confuses readers. ΔWTP^hat stands for “the monetized value of WTP changes”, so some other notation will be better. | We sincerely appreciate your pointing out the problem with the symbol representation. As you mentioned, the representation of ΔWTP^hat (it should actually be ΔSWB^hat, perhaps there was an error in the original text) is indeed prone to causing confusion among readers, and we have fully recognized this. Based on your suggestion, in order to make the expression clearer, and considering that we no longer calculate the willingness to pay (WTP) based on the predicted subjective well-being (SWB), we have changed ΔSWB^hat to Value (∆SWB). Such a change can more intuitively reflect its meaning of the monetized value of the change in SWB, avoiding ambiguity due to symbol issues, thereby helping readers better understand our research content and relevant analyses.  We will ensure that all relevant parts of the paper use the new symbol uniformly, thereby guaranteeing the consistency and readability of the paper's expression. |
| 9) Data availability statement states only the URL of the CHIP (which I could not access for some reason), but the authors used various other datasets. Further, these data sources are not cited in the reference (CHNS, NASA’s satellite data, etc). They should be cited. Data availability and reasons that data cannot be shared by the authors should be more clearly stated, instead of just writing the URL for CHIP dataset. | We sincerely appreciate your detailed and valuable review comments. We sincerely admit that when writing the manuscript, we neglected to accurately cite some of the key datasets used in the references. We not only used the CHIP dataset but also other important datasets such as CHNS and NASA’s satellite data, which played an indispensable supporting role in our research.  For the CHNS data, we added the following to the Data availability statement: This research uses data from China Health and Nutrition Survey (CHNS). We thank the National Institute of Nutrition and Food Safety, China Center for Disease Control and Prevention, Carolina Population Center, the University of North Carolina at Chapel Hill, the NIH (R01-HD30880, DK056350, and R01-HD38700) and the Fogarty International Center, NIH for financial support for the CHNS data collection and analysis files from 1989 to 2006 and both parties plus the China-Japan Friendship Hospital, Ministry of Health for support for CHNS 2009 and future surveys.  For the NASA’s satellite data, which were used to track sulfur dioxide concentrations across regions, providing spatial granularity for analysis. we added the following to the reference section:  Global Modeling and Assimilation Office (GMAO) (2015), inst3_3d_asm_Cp: MERRA-2 3D IAU State, Meteorology Instantaneous 3-hourly (p-coord, 0.625x0.5L42), version 5.12.4, Greenbelt, MD, USA: Goddard Space Flight Center Distributed Active Archive Center (GSFC DAAC), Accessed Enter User Data Access Date at doi: 10.5067/VJAFPLI1CSIV.  Finally, we uploaded the minimum anonymous dataset that can reproduce the research results to the public database Zenodo and clearly marked the data source. The DOI of the dataset is: 10.5281/zenodo.14556862. |
| 10) Language editing is needed. While overall the manuscript is written in good English, there are quite a lot of small mistakes (e.g. German instead of Germany). Wordy and lengthy parts can be shorten. | We sincerely appreciate your taking the time to review our manuscript and pointing out the language problems. We have fully recognized the necessity of language editing and have taken the following measures to improve:  (1) We carefully checked the manuscript for small errors such as misspelling "Germany" as "German". We screened the text sentence by sentence, using professional grammar checking tools and cross-checking among team members to ensure that all such minor errors in words, grammar, etc. were comprehensively corrected.  (2) For the wordy and lengthy parts of the manuscript, we carefully sorted them out, re-examined the structure of each sentence and paragraph, and deleted redundant, repetitive or unnecessary modifying sentences while ensuring the complete conveyance of the core viewpoints, so as to improve the readability and conciseness of the article. |
