# Supplementary material for: Wealth, health, and happiness: An inverse story of the Easterlin Paradox in China
Source: PLoS One. 2026 Mar 12;21(3):e0342445. doi: 10.1371/journal.pone.0342445 (PMC12981521; doi:10.1371/journal.pone.0342445)
Supplement: Attachment — Submitted filename: Comments.docx [file pone.0342445.s003.docx]

The authors made a significant improvement to the manuscript. The methods are improved, and the results are presented in a logically organized manner compared to the previous manuscript.

Whereas I appreciate the improvement made by the authors, I still see several points that need to be clarified. Some of the points are related to methodologies, while some others are related to inaccurate, confusing explanations (and sometimes explanations are lacking, old sentences from the previous manuscript are remaining, etc).

Why don’t you use FE or RE for CHNS dataset?

- The authors noted that CHNS data is a panel dataset. They further stated “*the longitudinal essential of the data also allows us to better control for unobserved individual heterogeneity and the potential selection bias problem*” in Section 4.1. However, based on the methods and results, the authors seemingly did not use individual/household FE to control these confounding factors. If there is a reason for not using the individual fixed effects and treating the CHNS panel as just a pooled dataset, then the authors should describe so and justify it. The authors also need to delete some of the sentences that sounds as if the authors are using FEs. Otherwise, it is straightforward to use individual FEs.

Data description

- Apart from the comment above, the description of the data sources is confusing. The authors wrote “*We combine data from the following sources to generate a 6 years panel across 8 provinces, 40 cities (21 cities are TCZ cities and 19 are non-TCZ cities), 5,058 households and 19,538 individuals to accomplish our research targets*” at the beginning of Section 4.1. But this is simply inaccurate. Firstly, the estimations based on the CHNS data have the sample sizes around 23,000 (Table 3), whereas those based on the CHIP data have the sample size of 20,887 (Table 4). Secondly, the area coverage and timing of these surveys are different. CHNS cover 10 provinces, whereas CHIP cover all provinces.
- As the authors use two different datasets, the authors should describe the sample size and data coverage of each dataset separately.

IV regression between SWB, SO2 and wage (Table 4)

- Firstly, I would like to confirm this point. In Table 4, only IVs and instrumented endogenous variables are shown. Did the authors use other variables but omit them from the table? The estimation equations (3)-(5) in Section 4.2 include other variables. Or did the authors actually not use any other variables? In other tables, the authors put notes like “Individual control YES”, so it seemed that the authors did not use any other variable in Table 4. I made the following comments, assuming that the authors did not use any other variables.
- Then, the use of these IVs can have several problems. Thermal inversion and wind speed themselves can be nice IVs, but they are likely to be correlated to geographical characteristics (thermal inversion is generally more frequent in cold areas and wind speed can be stronger in, say, coastal areas). These characteristics are likely to be correlated to the local economic conditions and people’s SWB. So, to use these IVs, the city-level and province level characteristics, such as weather conditions and development levels, need to be controlled for.
- The average income as an IV is more questionable. Firstly, living in a high-income area itself can have a direct influence on SWB. Secondly, the average income can cause a residential sorting problem. For example, a high wage area can attract economy-oriented, young and high-skilled individuals who value monetary aspects of SWB greatly. Then the second-stage coefficient of the wage can reflect the effect of such residential sorting, not a pure effect of income on SWB.
- Thus, to use these IVs as reliable exogenous factors, I suppose that the authors need to control for potential confounding factors. If the authors actually used other control variables and forgot to mention so, it is fine, but the coefficients of other variables should be provided. If the authors did not use any control variables, then they should add some variables and check if the results remain unchanged.

A related question regarding the IV regression (Table 4)

- Why is the sample size in column 1 just 4,224? It must be equal to the second stage result. Did you separately estimate these three equations?

Estimation of wages (Table 3)

- The authors regarded the column 4 as their main result and used it in the calculation of WTP and the monetary benefit of the TCZ policy. However, I wonder if the column 4 is the best specification.
- Firstly, the coefficient of the TCZxPost in column 4 (-26.089) is quite different from those in the other three columns (-15.075 to -16.580). I wonder if the column 4 coefficient is robust.
- The difference between column 4 and 3 is the addition of lnGDP. There is no detailed explanation for the definition of this variable, but I suppose that it is the city-level GDP (because the national GDP cannot be used with the year FE, and the province level GDP cannot be used with the province-year FE). Whatever the definition is, however, it is very counterintuitive that this variable has a negative coefficient on wage. It should be positive, at least theoretically. Is there a possibility that lnGDP is causing a multi-collinearity problem and consequently the coefficient of TCZxPost changed greatly?
- I actually do not think that lnGDP is particularly necessary because the authors have already controlled for the year FE, city FE and Province-Year FE. So it is likely that lnGDP encounters multi-collinearity with these FEs. In addition, the authors can use individual FE for this estimation. I recommend the authors to drop column 4 and use column 3 as the main results to calculate WTP, after adding individual FEs.

Parallel trend test for wage (Table 5)

- The authors claim that the coefficient of TCZ*fake policy timing is insignificant and thus the parallel trend is supported. But, after all, the magnitude of the coefficient itself is almost unchanged from Table 3. The standard errors increased a bit, and this made the coefficients insignificant, but the increased standard errors can be just a reflection of the decreased sample size. I think it is weak as evidence for parallel trend.
- I actually wonder why the authors did not draw a figure like Figure 3. The authors state “traditional parallel trend figure is not available, since the analysis is done using survey data which has limited number of periods to graph such figure”. But the CHNS survey was conducted in 1991, 1993, 1997, 2000, 2004, and 2006. It seems sufficient to draw a figure.

Year-by-year comparison starting from Table 9

- The authors noted that year 2002 is a bit different from other years, with a few times larger WTP. The authors noted either the possibility of structural changes occurring after 2002 or the possibility that 2002 is just an outlier. But both beta^hat and gamma^hat are insignificant (particularly gamma^hat is very small) in 2002. It is not really meaningful to assess the specialty of year 2002.
- Based on this point, “Using only 2002 result” in Tables 10 and 13 could be dropped.

Comparison with existing literature

- I noticed that the authors added a few citations in the literature review based on the comment from the other reviewer, such as: “*Abundant research also studied the effect of TCZ policy. However, due to data limitations, most of the existing literature have to choose focusing on a single aspect of the policy effects. For example, Zheng et al. (2005), Huang et al. (2022), Wang et al. (2023), and Yang et al. (2023) all focus on the pollution reduction or health effect of the policy, while Sun et al. (2019) and Li et al (2024) focus on the policy shock to income.*” But the authors should also compare their own results with these studies, maybe when showing results. WTP may not be comparable to these studies, but the authors’ results on pollution reduction, health effects, and effects on income could be compared to these studies. Were the authors’ results consistent with these studies, better than them, or over- or under-estimating the effects?

Variable definitions

- Definitions of some of variables are not provided.
- Perhaps the biggest point is wage. I do not see any definition of this variable. Is it daily, weekly, monthly, or annual? Is it the total wage income or the wage rate? Also I wondered why the mean values of “Wage” and “Annual household income” are so different. For example, the mean of wage is 205.2 and that of annual household income is 20.13 in the CHNS data. Given that the definitions of these variables are not provided and their unit is not clear, it is confusing.
- Definitions of control variables should also be provided. For example, nationality (dummy variable taking the value of one if an individual is Chinese and zero otherwise?) and gender (the mean value is 1.529 so 1 if male and 2 if female?) are used without any explanation. The meaning of GDP is not provided either. The national GDP cannot be used with the year fixed effects so I suppose that this variable is actually city-level GDP (as I noted above). But anyway, its definition is not provided at all. What are Denc score and market score, what are the data sources for these variables, and what is the objective of the authors to use these variables?
- In Table 5 and below, terms like individual control, family control, etc are used. Individual, household, and weather controls are fine, but what are community control and city control?
- In sum, the definitions of variables should be explained in text, tables, or appendix.

Table 8

- It looks like that the authors ran two separate estimations. But based on the equation, I think that the authors ran a single regression and showed the marginal effects of Health2 in column (1) and those of Health3 in column (2). It is confusing, particularly because of the rows “Individual control” to “Observations.”
- The authors state that “*we find that compared with a resident who reports his\er health status to be “Fair”, being “Healthy” increased the probability of the resident to feel “Very Happy” by 10.7%.*” I don’t think it is correct. Based on the equation, the marginal effect of health3=1 on the probability to be fair (-0.107) must be the change in the probability compared to health1=1, so the 10.7% change in the probability is compared to a resident who reported the health status to be “Unhealthy.”
- Additionally, it should be “decreased”, not “increased” because the sign of the marginal effect is negative.
- This table and Table 7 need more clarification. After all, there is no explanation that these tables show marginal effects.

Comparability of CHNS and CHIP, and matching

- The authors noted that the area coverages of these two datasets are different and they checked this concern (below Table 11). So they checked if SO2, lnGDP, Temperature, and Rain are similar, using propensity score matching.
- I don’t see the point of this exercise. Firstly, if the similarity of the province/city-level characteristics is the only concern, as stated by the authors, then the authors can simply restrict the CHIP samples to the 10 provinces covered by the CHNS.
- Secondly, this exercise does not assure the similarity of these samples in terms of SWB, income, age, and other individual-level characteristics. If the authors employ PSM, then the similarity of these individual factors should be of the main interest.
- In addition, Tables 11 and 12 do not have any information of the observation size. So we do not know how many samples were dropped through the PSM process.

Need for more editing

- The authors replied that they “carefully checked the manuscript for small errors such as misspelling "Germany" as "German".” But I still see German instead of Germany…
- Well, this misspelling itself is a minor mistake, but there are several other grammatical and spelling errors remaining. E.g., “*However, the question remains is, will this […]*” in introduction, “*DGP*” instead of GDP in Table 1, and “*citizens of relative wealthier areas*” at the end of conclusion. In addition, as I commented a few of them above, the manuscript has several confusing, careless, and inaccurate sentences. The authors need to once more carefully check these details.
