# Supplementary material for: Wealth, health, and happiness: An inverse story of the Easterlin Paradox in China
Source: PLoS One. 2026 Mar 12;21(3):e0342445. doi: 10.1371/journal.pone.0342445 (PMC12981521; doi:10.1371/journal.pone.0342445)
Supplement: Attachment — Submitted filename: Response to Reviewers_V4.docx [file pone.0342445.s004.docx]

Dear Editor,

Thank you for your email of 4 April 2025 concerning our manuscript “Wealth, Health, and Happiness: An Inverse Story of the Easterlin Paradox in China” (PONE-D-23-32960). We are extremely grateful for the opportunity to revise our manuscript based on the reviewers' constructive feedback and your guidance. We have revised the paper according to the reviewer’s suggestions and have marked the changes in red.

The major revisions are summarized as follows:

1. In the model for estimating the TCZ effect, we incorporated individual fixed effects. This helps better control for unobserved individual heterogeneity, which was a methodological oversight in the initial submission. Moreover, we removed GDP as a control variable. Its inclusion led to instability and theoretical inconsistency in coefficient estimates, likely due to multicollinearity.
2. We conducted a parallel trend test for wages. This is an important step to ensure the validity of our difference-in-differences analysis. By doing so, we can more confidently draw conclusions about the causal impact of the TCZ policy on wages.
3. We carefully proofread the entire manuscript for grammar errors. We corrected a wide range of mistakes, including misspellings, incorrect variable notations, and improper sentence structures.

With this letter, we have resubmitted our manuscript which we would like to be considered for publication in PLOS ONE. In order to facilitate the review of our revisions, we are attaching a detailed, comment by comment response to the reviewer’s concerns.

If you need anything else, please do not hesitate to contact us.

Kind regards,

All authors

Response of Authors to the Comments of Reviewer #2

“Wealth, Health, and Happiness: An Inverse Story of the Easterlin Paradox in China”

| **Reviewer’s Comments** | **Authors’ Response** |
| --- | --- |
| Comments to the Author  GENERAL COMMENTS  The authors made a significant improvement to the manuscript. The methods are improved, and the results are presented in a logically organized manner compared to the previous manuscript.  Whereas I appreciate the improvement made by the authors, I still see several points that need to be clarified. Some of the points are related to methodologies, while some others are related to inaccurate, confusing explanations (and sometimes explanations are lacking, old sentences from the previous manuscript are remaining, etc). | Thank you very much for your constructive comments and suggestions. We have revised the manuscript carefully in response to your comments. We hope you find the revisions satisfactory. |
| MAJOR COMMENTS  1)Why don’t you use FE or RE for CHNS dataset?   - The authors noted that CHNS data is a panel dataset. They further stated “the longitudinal essential of the data also allows us to better control for unobserved individual heterogeneity and the potential selection bias problem” in Section 4.1. However, based on the methods and results, the authors seemingly did not use individual/household FE to control these confounding factors. If there is a reason for not using the individual fixed effects and treating the CHNS panel as just a pooled dataset, then the authors should describe so and justify it. The authors also need to delete some of the sentences that sounds as if the authors are using FEs. Otherwise, it is straightforward to use individual FEs. | Thank you very much for your insightful comment. We sincerely appreciate your suggestion regarding the use of individual fixed effects (FEs) for the CHNS dataset.  In our initial submission, we did not include individual fixed effects in the CHNS sample regressions, which may have led to potential concerns regarding unobserved heterogeneity. We acknowledge this as a methodological omission and have now addressed it in our revised manuscript. Specifically, we have re-estimated the relevant regressions using individual fixed effects, implemented via the “reghdfe” command in Stata. By including individual FEs, we are able to control for all time-invariant individual-specific characteristics, thereby improving the robustness of our estimates and better addressing potential selection bias.  In addition, as you rightly pointed out in your subsequent comment, we observed instability and theoretical inconsistency in the coefficient estimates of the GDP variable, which is likely due to multicollinearity. In response, we have removed GDP from all relevant equations to enhance the model’s clarity and focus.  Specifically, we add $\tau_{i}$which represents individual fixed effect in equation (2). We also revised the related descriptions in Section 4.1:  “$X_{ihcjpt}$ is a series of time-varying individual level control variables including years of education, age, and marital status. $M_{hcjpt}$ is a series of household level control variables including family size and the household’s income. $D_{cjpt}$ is a series of community level control variables including marketization score and transportation infrastructure score. $\tau_{i}$is the individual fixed effect”.  Thank you again for your constructive feedback, which has helped us significantly improve the methodological rigor of our work. |
| 2) Data description   - Apart from the comment above, the description of the data sources is confusing. The authors wrote “We combine data from the following sources to generate a 6 years panel across 8 provinces, 40 cities (21 cities are TCZ cities and 19 are non-TCZ cities), 5,058 households and 19,538 individuals to accomplish our research targets” at the beginning of Section 4.1. But this is simply inaccurate. Firstly, the estimations based on the CHNS data have the sample sizes around 23,000 (Table 3), whereas those based on the CHIP data have the sample size of 20,887 (Table 4). Secondly, the area coverage and timing of these surveys are different. CHNS cover 10 provinces, whereas CHIP cover all provinces. - As the authors use two different datasets, the authors should describe the sample size and data coverage of each dataset separately. | Thank you very much for pointing out the confusion in our data description. We appreciate your detailed and constructive comments. In response, we have made the following clarifications and revisions:  First, regarding the inconsistency in sample sizes, we acknowledge that our earlier description was unclear. For the CHNS-based estimations, the total number of observations used in our analysis is 27,561, covering 12,364 individuals from 4,632 households. Since CHNS is a longitudinal survey, the number of individuals is smaller than the total number of observations due to repeated measurements over time. For the CHIP-based estimations, the total number of observations is 22,199.  Second, we recognize that our original description inaccurately combined details from both datasets, which have different geographical coverage and time spans. CHNS covers selected provinces and provides panel data from 1991 to 2006, while CHIP includes broader geographic coverage and is based on pooled cross-sectional data from 2002, 2007, 2008, and 2013.  To correct this, we have revised the relevant paragraph on page 19. The original statement:  “We combine data from the following sources to generate a 6-year panel across 8 provinces, 40 cities (21 cities are TCZ cities and 19 are non-TCZ cities), 5,058 households and 19,538 individuals to accomplish our research targets.” has been replaced with: “In this research, we combine data from the following sources to accomplish our research targets.”  Details of the data coverage are moved to separate descriptions for each data source. More specifically, in the section introducing the CHNS data (page 21 of the revised manuscript), we add:  “The CHNS sample used in our analysis includes approximately 27,561 observations, covering 8 provinces and 40 cities (21 of which are TCZ cities and 19 are non-TCZ cities). It involves 4,632 households and 12,364 individuals, with data spanning from 1991 to 2006”.  On page 21 of the revised manuscript, which introduces the CHIP data, we add:  “The CHIP sample used in our analysis includes 22,199 observations, covering most regions across the country, with survey waves conducted in 2002, 2007, 2008, and 2013.”  We hope these clarifications address your concerns and improve the clarity and accuracy of our data description. Thank you again for your valuable suggestions. |
| 3) IV regression between SWB, SO2 and wage (Table 4)   - Firstly, I would like to confirm this point. In Table 4, only IVs and instrumented endogenous variables are shown. Did the authors use other variables but omit them from the table? The estimation equations (3)-(5) in Section 4.2 include other variables. Or did the authors actually not use any other variables? In other tables, the authors put notes like “Individual control YES”, so it seemed that the authors did not use any other variable in Table 4. I made the following comments, assuming that the authors did not use any other variables. | Thank you very much for your insightful comment and for pointing out the ambiguity in our table presentation.  We would like to clarify that the estimations reported in Table 4 were indeed conducted based on Equations (3), (4), and (5), and **all control variables** included in these equations were used in the regressions. These include individual-level controls (e.g., age, gender, marital status), household-level controls (e.g., household income, household size), and city-level controls (e.g., temperature, precipitation).  However, we acknowledge that we failed to explicitly indicate the inclusion of these control variables in the original version of Table 4, which may have led to the misunderstanding. To address this, we have revised the table by adding appropriate notes: “Individual controls: YES; Household controls: YES; Weather controls: YES; Year FE: YES; City FE: YES; Province-by-year FE: YES” to clarify that these variables were included in the estimation. We have also presented the regression results of all control variables’ coefficients in Table A1 in the Appendix.  We appreciate your careful reading and helpful suggestions, and we believe these changes will improve the clarity and transparency of our presentation. |
| - Then, the use of these IVs can have several problems. Thermal inversion and wind speed themselves can be nice IVs, but they are likely to be correlated to geographical characteristics (thermal inversion is generally more frequent in cold areas and wind speed can be stronger in, say, coastal areas). These characteristics are likely to be correlated to the local economic conditions and people’s SWB. So, to use these IVs, the city-level and province level characteristics, such as weather conditions and development levels, need to be controlled for. | Thank you very much for raising this important concern regarding the validity of our instrumental variables.  To address the potential endogeneity arising from the correlation between thermal inversion, wind speed, and underlying geographical or socioeconomic characteristics, we have taken the following steps:  1) Control City Fixed Effects: We include city fixed effects in all IV regressions to account for time-invariant city-level characteristics, such as geography, location (e.g., coastal vs. inland). This helps to mitigate the concern that differences in geography or structural development could bias the IV estimates.  2) Include Province-Year Fixed Effects: To control for time-varying regional shocks and macroeconomic conditions that may influence both air pollution and SWB, we further include province-by-year fixed effects. This controls for province-level trends and annual shocks such as economic policy changes, infrastructure investment, or regional development initiatives.  3) Time-varying city level controls: In addition, we include time-varying weather variables (e.g., temperature, precipitation) at the city level to further mitigate any residual confounding related to short-term weather fluctuations that may affect both pollution levels and well-being.  We believe these strategies jointly address the concern of omitted variable bias and support the validity of our IV approach. Thank you again for your thoughtful and constructive feedback. |
| - The average income as an IV is more questionable. Firstly, living in a high-income area itself can have a direct influence on SWB. Secondly, the average income can cause a residential sorting problem. For example, a high wage area can attract economy-oriented, young and high-skilled individuals who value monetary aspects of SWB greatly. Then the second-stage coefficient of the wage can reflect the effect of such residential sorting, not a pure effect of income on SWB. | Thank you very much for your insightful comments regarding the use of provincial-level average industry wage as an instrumental variable for individual income.  We acknowledge the concerns that (1) average income may directly affect SWB, and (2) it may lead to residential sorting, thus violating the exclusion restriction. To address these issues, we have included a range of control variables in our model, including age, education level, marital status, household income, and household size. We apologize for not making this sufficiently clear in the previous version of the table.  In response to your comments, we have further included additional controls such as self-reported health status, city-level population size to better account for potential confounding effects related to regional development and individual health conditions. The updated results, reported in Table A2 of the Appendix, remain consistent with our baseline findings, suggesting that our main conclusions are robust to these concerns.  We greatly appreciate your constructive feedback, which helped us strengthen the empirical validity of our analysis. |
| - Thus, to use these IVs as reliable exogenous factors, I suppose that the authors need to control for potential confounding factors. If the authors actually used other control variables and forgot to mention so, it is fine, but the coefficients of other variables should be provided. If the authors did not use any control variables, then they should add some variables and check if the results remain unchanged. | Thank you very much for pointing out the importance of controlling for potential confounding factors when using instrumental variables (IVs).  We would like to clarify that we did include a comprehensive set of control variables in the IV regressions, but we apologize for not stating this clearly in the previous version of the manuscript. Specifically, we controlled for a variety of individual- and household-level characteristics, such as age, education, marital status, household income, and household size, to mitigate possible confounding effects. At the city level, we controlled for city fixed effects to account for time-invariant geographical and policy-related factors, as well as city-specific weather conditions (e.g., temperature and precipitation). In addition, we included province-by-year fixed effects to capture broader macroeconomic and environmental variations across time and regions.  Although these controls were included in the model estimation, we did not clearly report them in the table notes or results. In the revised version, we have now explicitly indicated the inclusion of control variables in the table captions and added the estimated coefficients of these controls in the appendix for transparency and completeness.  We greatly appreciate your careful review and helpful suggestions. We believe these revisions significantly improve the clarity and credibility of our empirical strategy. |
| 4) A related question regarding the IV regression (Table 4)   - Why is the sample size in column 1 just 4,224? It must be equal to the second stage result. Did you separately estimate these three equations? | Thank you very much for your careful review.  The reason why the sample size in column 1 is different from that in column 3 is because the endogenous variable $P_{jpt}$ (which is measured by SO_2_ concentration in city $j$ of province $p$ at time $t$) itself is a city level observation, while the independent variable ${SWB}_{ihjkpt}$ in the 2^nd^ stage regression is an individual level measurement associated with individual $i$ from household $h$. As a result, in the 1^st^ stage regression regarding $P_{jpt}$(see Equation (3)), the observation is just 4,224, while in the 2^nd^ stage regression regarding ${SWB}_{ihjkpt}$(see Equation(5)), the observation is 22,199. In comparison, since the endogenous variable $lnR_{ihjkpt}$ is also an individual variable associated with individual $i$ from household $h$, in the 1^st^ stage regression regarding this variable (see Equation (4) and Column (2)), the observation is 22,199, equals to that in the 2^nd^ stage regression. To match the hierarchical structure of the data, we estimated the 2SLS IV model in two separate steps.  We appreciate your attention to this technical detail and hope this explanation clarifies the issue. |
| 5) Estimation of wages (Table 3)   - The authors regarded the column 4 as their main result and used it in the calculation of WTP and the monetary benefit of the TCZ policy. However, I wonder if the column 4 is the best specification. - Firstly, the coefficient of the TCZxPost in column 4 (-26.089) is quite different from those in the other three columns (-15.075 to -16.580). I wonder if the column 4 coefficient is robust. - The difference between column 4 and 3 is the addition of lnGDP. There is no detailed explanation for the definition of this variable, but I suppose that it is the city-level GDP (because the national GDP cannot be used with the year FE, and the province level GDP cannot be used with the province-year FE). Whatever the definition is, however, it is very counterintuitive that this variable has a negative coefficient on wage. It should be positive, at least theoretically. Is there a possibility that lnGDP is causing a multi-collinearity problem and consequently the coefficient of TCZxPost changed greatly? - I actually do not think that lnGDP is particularly necessary because the authors have already controlled for the year FE, city FE and Province-Year FE. So it is likely that lnGDP encounters multi-collinearity with these FEs. In addition, the authors can use individual FE for this estimation. I recommend the authors to drop column 4 and use column 3 as the main results to calculate WTP, after adding individual FEs. | Thank you very much for your professional and meticulous comments regarding the estimation of the TCZ policy’s impact on wages.  We fully agree with your concerns about the robustness of the results presented in Column (4). As you rightly pointed out, the coefficient of TCZ × Post in Column (4) (−26.089) deviates notably from the estimates in Columns (1)–(3) (−15.075 to −16.580), raising valid concerns about the stability of the results.  The primary difference between Columns (3) and (4) is the inclusion of the variable lnGDP. We acknowledge that lnGDP is likely to be highly collinear with the fixed effects already included in the model, i.e., the year fixed effects, the city fixed effects, and the province year fixed effects. This multicollinearity could indeed explain both the unexpected sign of the lnGDP coefficient (negative, whereas a positive relationship with wage is expected) and the large shift in the estimated coefficient of TCZ×Post.  Moreover, as you noted, given the presence of those fixed effects, lnGDP may not provide substantial additional explanatory power, and including it could do more harm than good in terms of estimation precision. Therefore, based on your suggestion and our own reconsideration, we have removed lnGDP from the model, and instead incorporated individual fixed effects to further improve the estimation.  We have re-estimated the regressions accordingly and updated Table 3 in the revised manuscript. We now regard the specification in Column (3), augmented with individual fixed effects, as our preferred model for the calculation of WTP and the monetary benefit of the TCZ policy.  Thank you again for your insightful and constructive feedback. We believe these changes significantly enhance the robustness and credibility of our findings. |
| 6) Parallel trend test for wage (Table 5)   - The authors claim that the coefficient of TCZ*fake policy timing is insignificant and thus the parallel trend is supported. But, after all, the magnitude of the coefficient itself is almost unchanged from Table 3. The standard errors increased a bit, and this made the coefficients insignificant, but the increased standard errors can be just a reflection of the decreased sample size. I think it is weak as evidence for parallel trend. - I actually wonder why the authors did not draw a figure like Figure 3. The authors state “traditional parallel trend figure is not available, since the analysis is done using survey data which has limited number of periods to graph such figure”. But the CHNS survey was conducted in 1991, 1993, 1997, 2000, 2004, and 2006. It seems sufficient to draw a figure. | Thank you very much for your valuable comments regarding the parallel trend test. We fully acknowledge your concern that the previous evidence presented in Table 5 is insufficient to robustly support the parallel trend assumption.  In response to your suggestion, we have re-evaluated the feasibility of constructing a traditional parallel trend figure. While we previously noted that the CHNS data had a limited number of waves, we now realize that there are sufficient survey rounds (1991, 1993, 1997, 2000, 2004, and 2006) to create a meaningful event-study-style plot. Accordingly, we have now drawn a new figure (Figure 4) that visualizes the dynamics of the wage trend between the treatment and control groups over time, using data from the aforementioned survey years. We also include individual fixed effects in this analysis to better account for unobserved heterogeneity.  This figure provides a more intuitive and compelling visual check for the parallel trend assumption. We have added this new figure to page 34 of the revised manuscript, along with a corresponding discussion in the main text. Specifically, we write on page 34:  “Similarly, Figure 4 presents the parallel trend test results for the effect of the TCZ policy on wages, i.e., equation (2). Again, the difference between the two groups is insignificant prior to the policy, while it becomes significant afterward, further supporting the credibility of our DID identification strategy.”  Thank you again for your insightful and constructive suggestions. We believe these revisions strengthen the credibility and transparency of our empirical strategy. |
| 7) Year-by-year comparison starting from Table 9   - The authors noted that year 2002 is a bit different from other years, with a few times larger WTP. The authors noted either the possibility of structural changes occurring after 2002 or the possibility that 2002 is just an outlier. But both beta^hat and gamma^hat are insignificant (particularly gamma^hat is very small) in 2002. It is not really meaningful to assess the specialty of year 2002. - Based on this point, “Using only 2002 result” in Tables 10 and 13 could be dropped. | Thank you very much for your valuable comments on the year-by-year comparison.  We agree with your analysis of the results in 2002. Accordingly, in the revised manuscript, we have deleted the content in the third column corresponding to “Using only 2002 result” in Tables 9 and 12. Please note that Table 10 and Table 13 in the original version have been renumbered as Table 9 and Table 12, respectively, following the replacement of the original Table 5 (which reported the parallel trends in wages) with the new Figure 4. This adjustment helps avoid presenting potentially misleading or statistically insignificant results. It also contributes to a more concise and rigorous presentation of the findings.  During the revision process, we carefully reviewed the relevant sections to ensure that the logical coherence and analytical integrity of the paper remained intact after this deletion. Specifically, on pages 39 - 40, the description of Table 9 has been revised as follows:  “First, we use the average ratio of the 2007 - 2013 estimates to approximate the ratio in the policy shock year (the average scenario in Table 9). Second, a linear interpolation approach is employed, predicting the ratio in the policy shock year to be 0.79 (the linear interpolation scenario in Table 9). For the second component, we adopt the average income of subsamples from the affected areas in CHNS data. The related results are reported in Table 9.”  Thank you again for your professional suggestions, which have been of great help to us in improving the paper. |
| 8) Comparison with existing literature   - I noticed that the authors added a few citations in the literature review based on the comment from the other reviewer, such as: “Abundant research also studied the effect of TCZ policy. However, due to data limitations, most of the existing literature have to choose focusing on a single aspect of the policy effects. For example, Zheng et al. (2005), Huang et al. (2022), Wang et al. (2023), and Yang et al. (2023) all focus on the pollution reduction or health effect of the policy, while Sun et al. (2019) and Li et al (2024) focus on the policy shock to income.” But the authors should also compare their own results with these studies, maybe when showing results. WTP may not be comparable to these studies, but the authors’ results on pollution reduction, health effects, and effects on income could be compared to these studies. Were the authors’ results consistent with these studies, better than them, or over- or under-estimating the effects? | Thank you very much for your thoughtful suggestions regarding the comparison with existing literature. However, due to significant differences in the units of analysis and research scopes, a direct comparison of the estimated values is not feasible. While the existing studies primarily examine the policy impacts at the city level, our analysis is conducted at the individual level, making the numerical results not directly comparable. However, we do offer a series of comparisons on the direction of our results against these papers. Specifically, we added the following statements:  On page 29: “This finding is consistent with Huang et al. (2022), who also report that the TCZ policy contributes to a decline in SO₂ emissions.”  On page 30: “Sun et al. (2019) also show that the TCZ policy reduces the average income in small and medium-sized cities.”  On page 37: “which is consistent with the research findings of Wang et al (2023). Specifically, their study found that the TCZ policy led to a 39% reduction in the four-week prevalence of diseases associated with air pollution.”  Thank you again for your helpful suggestions, which have enhanced the depth of our comparison with existing research. |
| 9) Variable definitions   - Definitions of some of variables are not provided. - Perhaps the biggest point is wage. I do not see any definition of this variable. Is it daily, weekly, monthly, or annual? Is it the total wage income or the wage rate? Also I wondered why the mean values of “Wage” and “Annual household income” are so different. For example, the mean of wage is 205.2 and that of annual household income is 20.13 in the CHNS data. Given that the definitions of these variables are not provided and their unit is not clear, it is confusing. | Thank you very much for pointing out the insufficient clarity in our variable definitions. We acknowledge that the lack of clear definitions for certain variables may have caused confusion in interpreting the results, and we sincerely apologize for this oversight.  To prevent similar confusion, we have revised Table 1 to include clearer definitions for all key variables, ensuring that readers can immediately understand their meaning and measurement units.  Specifically, “wage” refers to the average monthly salary of an individual, measured in Chinese yuan (CNY). In contrast, “annual household income” denotes the total annual income of a household, measured in thousand yuan. It is precisely because of the differences in measurement units and statistical objects between these two variables that their mean values appear to be quite different.  Thank you again for your careful review and constructive suggestions. We believe that these revisions enhance the transparency and clarity of our empirical analysis. |
| - Definitions of control variables should also be provided. For example, nationality (dummy variable taking the value of one if an individual is Chinese and zero otherwise?) and gender (the mean value is 1.529 so 1 if male and 2 if female?) are used without any explanation. The meaning of GDP is not provided either. The national GDP cannot be used with the year fixed effects so I suppose that this variable is actually city-level GDP (as I noted above). But anyway, its definition is not provided at all. What are Denc score and market score, what are the data sources for these variables, and what is the objective of the authors to use these variables? | Thank you very much for highlighting the lack of clarity in the definitions of our control variables. We have added detailed definitions for these variables in Table 1 on page 22. Regarding the concern you mentioned in this comment:  “nationality” is a dummy variable which takes a value of 1 when the individual observed belongs to the Han ethnicity, while a value of 0 represents an ethnic minority.  “gender” is a dummy variable which takes a value of 1 when the individual observed belongs to male, while a value of 0 represents female.  “GDP” which referred to city-level GDP, has been removed in the revised manuscript to avoid potential multicollinearity.  “Denc score” and “Market score”, are community-level variables derived from the CHNS dataset. The “Denc score” captures the population density of the community, while the “market score” reflects the degree of marketization in the local area. These variables are included to control for contextual factors at the community level, thereby improving the accuracy and robustness of our estimations.  Thank you again for your insightful suggestions, which have greatly helped us improve the clarity and rigor of the manuscript. |
| - In Table 5 and below, terms like individual control, family control, etc are used. Individual, household, and weather controls are fine, but what are community control and city control? - In sum, the definitions of variables should be explained in text, tables, or appendix. | Thank you very much for your attention and feedback regarding the issues related to variable definitions. We have added detailed definitions for all key variables in Table 1 on page 22, including independent variables, dependent variables, and various control variables. In the table, we have explained in detail the name, definition, and value range (if it is a categorical variable) of each variable. Regarding the concern you mentioned in this comment:  The term community control refers to variables at the community level, specifically the transport score and the market score, both derived from the CHNS dataset. The transport score reflects the transportation infrastructure score within a community, while the market score captures the extent of marketization and development of the local economic environment. The term city control previously referred to city-level GDP, which has been removed from the revised analysis to avoid multicollinearity with fixed effects.  Thank you again for your professional suggestions. |
| 10) Table 8  - It looks like that the authors ran two separate estimations. But based on the equation, I think that the authors ran a single regression and showed the marginal effects of Health2 in column (1) and those of Health3 in column (2). It is confusing, particularly because of the rows “Individual control” to “Observations.” | Thank you very much for your attention to Table 8. As you inferred, we actually estimated one equation. First, we used the ologit model for estimation. This model can effectively handle the relevant data and variable relationships involved in our research. After completing the estimation with the ologit model, we calculated the related marginal effects with the post-estimation command “margin” provided by the package to give more straight forward and meaningful results.  The confusion may have arisen from the way we presented the marginal effects separately for Health2 and Health3 in different columns for clarity. In the rows from “Individual control” to “Observations”, the data actually corresponds to the results under the same regression model. When presenting the marginal effects, in order to allow readers to intuitively compare the marginal effects of Health2 and Health3, we adopted a column-separated form, but this may have masked to some extent the fact that they come from the same regression.  To eliminate this confusion, we have reported the original result in Table A3 in the Appendix and revised the content of Table 7. In the updated Table 7 (formerly Table 8), the first column reports the predicted probability differences between individuals reporting “fair” and “poor” health. The second column presents the same for “good” versus “poor” health, and the third for “good” versus “fair” health. We also removed redundant rows (“Individual control” to “Observations”) and added a descriptive note to page 37 clarifying the table’s content:  “The original results of the regression can be found in Table A3 in the Appendix. Table 7 presents the marginal effects of reporting fair health (relative to poor health) and good health (relative to poor or fair health) on the probabilities of being very unhappy, unhappy, fair, happy, and very happy.”  Thank you again for your professional suggestions. We believe that through these improvements, the overall logic and readability of the paper will be further enhanced. |
| - The authors state that “we find that compared with a resident who reports his\er health status to be “Fair”, being “Healthy” increased the probability of the resident to feel “Very Happy” by 10.7%.” I don’t think it is correct. Based on the equation, the marginal effect of health3=1 on the probability to be fair (-0.107) must be the change in the probability compared to health1=1, so the 10.7% change in the probability is compared to a resident who reported the health status to be “Unhealthy.” - Additionally, it should be “decreased”, not “increased” because the sign of the marginal effect is negative. | Thank you very much for your careful review of the paper. After careful rechecking, we realized that the confusion may stem from the way the marginal effects were presented and interpreted in our manuscript. Specifically, the statement in question refers to the marginal effects reported in the fifth row of Table 7 (formerly Table 8).  Based on our model results, individuals reporting “good” health are 10.6 percentage more likely to report being “very happy” than those reporting “fair” health (0.186 vs. 0.083), as shown in the fifth row of Table 7. To clarify this point, we have revised the text on page 38 to: “As shown in Column (3) of the table, compared to those who report fair health, individuals in good health are 10.6 % more likely to report feeling ‘Very Happy’.”  Thank you again for your rigorous review. |
| - This table and Table 7 need more clarification. After all, there is no explanation that these tables show marginal effects. | Thank you very much for pointing out the problems in the expressions of Table 7(now Table 6) and Table 8(now Table 7). We fully accept your suggestions and immediately made the following revisions to the paper:  In the relevant sections of the paper, we added clear explanations to emphasize that these two tables show marginal effects. Specifically, we added the following clarifying statements above the tables on page 36 and page 37:  “Table 6 reports the marginal effects of the TCZ policy on the probabilities of being unhealthy, fair, and healthy, estimated from ordered logit models with progressively added sets of controls”  “Table 7 presents the marginal effects of reporting fair health (relative to poor health) and good health (relative to poor or fair health) on the probabilities of being very unhappy, unhappy, fair, happy, and very happy, based on an ordered logit model that includes individual, household, and weather-related covariates, as well as fixed effects.”  In addition, we changed the title of Table 7 (now Table 6) from “The Impact of Regulation on Health” to “The Marginal Effect of Regulation on Health,” and the title of Table 8 (now Table 7) from “Relationship between SWB and Health” to “The Marginal Effect of Health on SWB.”  Through these revisions, we hope to significantly improve the clarity and readability of the paper, ensuring that readers can accurately understand the information conveyed by these two tables.  Thank you again for your professional suggestions, which have played a crucial role in helping us improve the paper. |
| 11) Comparability of CHNS and CHIP, and matching  - The authors noted that the area coverages of these two datasets are different and they checked this concern (below Table 11). So they checked if SO2, lnGDP, Temperature, and Rain are similar, using propensity score matching. - I don’t see the point of this exercise. Firstly, if the similarity of the province/city-level characteristics is the only concern, as stated by the authors, then the authors can simply restrict the CHIP samples to the 10 provinces covered by the CHNS. - Secondly, this exercise does not assure the similarity of these samples in terms of SWB, income, age, and other individual-level characteristics. If the authors employ PSM, then the similarity of these individual factors should be of the main interest. - In addition, Tables 11 and 12 do not have any information of the observation size. So we do not know how many samples were dropped through the PSM process. | Thank you very much for your valuable comments on the comparability and matching of the CHNS and CHIP datasets in our research. In response to your questions, we provide the following replies:  First, regarding your suggestion about restricting the sample range based on provincial/city-level characteristics, we agree that this approach is more straightforward and effective. However, this would reduce the CHIP sample size to 8,576—meaning that more than half of the original observations would be lost—and the resulting estimates become statistically less robust. Therefore, we employed the Propensity Score Matching (PSM) method to include samples from other provinces whose provincial or city-level characteristics closely resemble those of the CHNS regions.  Second, with PSM, our primary goal is to ensure consistency in the external environments of the research subjects, thereby minimizing confounding effects from differing regional conditions. To this end, we mainly used city-level variables for matching, so that the results based on micro data remain contextually meaningful and interpretable.  Finally, after PSM, the final matched sample included 187 cities, while 27 cities were excluded during the matching process. We also report the statistics regarding the effectiveness of the PSM match in Table 10 on page 40. As shown in the table, the p-values from the t-tests for pollution levels in the matched samples exceed 0.1, indicating no statistically significant differences between the matched city samples and the cities in CHNS. Obviously, after the PSM matching, the similarity between the treated group and the control group has been significantly improved. We have also updated the main text to clarify the rationale and results of this exercise. The revised paragraph (pages 40–41) now reads as follows:  “As mentioned in the data section, while the CHIP data covers most provinces of mainland China, the CHNS data only covers 8 provinces. This raises concerns that the WTP estimated from the CHIP data may not accurately reflect the preferences of residents in the more limited CHNS data. To address this issue—and given that limiting the CHIP sample to the eight CHNS provinces would substantially reduce the sample size—we apply the propensity score matching (PSM) method to identify subsamples in the CHIP data that are similar to those of the CHNS data in terms of pollution levels and weather conditions. We adopt kernel matching with a bandwidth of 0.05, resulting in 187 matched city samples. Table 10 presents the evaluation of matching effectiveness. As shown in the table, the p-values from the t-tests for pollution levels in the matched samples exceed 0.1, indicating no statistically significant differences between the matched city samples and the cities in CHNS.”  Thank you again for your professional guidance. We look forward to your approval of these modifications. |
| 12) Need for more editing  - The authors replied that they “carefully checked the manuscript for small errors such as misspelling "Germany" as "German". ”But I still see German instead of Germany… - Well, this misspelling itself is a minor mistake, but there are several other grammatical and spelling errors remaining. E.g., “However, the question remains is, will this […]” in introduction, “DGP” instead of GDP in Table 1, and “citizens of relative wealthier areas” at the end of conclusion. In addition, as I commented a few of them above, the manuscript has several confusing, careless, and inaccurate sentences. The authors need to once more carefully check these details. | Thank you sincerely for your careful re-reading of our manuscript and for your valuable comments regarding the remaining language issues. We deeply apologize for the oversight and for failing to ensure that the manuscript met the expected language standards prior to submission. We take this issue very seriously.  In response, we have now thoroughly re-examined the entire manuscript for spelling, grammatical, and stylistic issues. Specifically, we have made the following corrections:  The repeated typo “German” has been uniformly corrected to “Germany”.  In the introduction, the sentence “However, the question remains is, will this […]” has been revised to “However, the question remains whether this regulation will […]”.  The typo “DGP” has been corrected to “GDP”.  In the conclusion, “citizens of relative wealthier areas” has been revised to “especially those living in industrialized and wealthier areas”.  In addition to these, we carefully proofread the manuscript paragraph by paragraph and corrected the following errors:  “off-set” → “offset” (p.3, line 15)  “traditional idea beliefs” → “traditional beliefs” (p.9, line 10)  “accompanied with” → “accompanied by” (p.10, line 4)  “wealth cumulation” → “wealth accumulation” (p.10)  “insight on” → “insight into” (p.10, line 9)  “cross-section data” → “cross-sectional data” (p.11, line 3)  “to research” → “in their analysis” (p.11)  “more easily to suffer” → “more prone to” (p.11)  “interviewers” → “interviewees” (p.11, line 15)  “over the year 2002–2013” → “over the years 2002 to 2013” (p.11, line 20)  “miracle economic growth” → “miraculous economic growth” (p.13, line 3)  “burn coals” → “burn coal” (p.15, line 12)  “a principal guidance” → “a principal guide” (p.15, line 18)  “completed” → “complete” (p.23, line 2)  “instrument variable” → “instrumental variable” (p.26, line 8)  “staffs” → “staff” (p.27, line 3)  “Column” → “Columns” (p.31, line 6)  “two IV” → “two IVs” (p.31, line 7)  “two estimate” → “two estimates” (p.31, line 10)  “pre-existed” → “pre-existing” (p.32, line 13)  We have now conducted a comprehensive proofreading of the manuscript, ensuring that the language is accurate, clear, and professional. We are truly grateful for your rigorous feedback, which has helped us greatly improve the overall quality of our paper. |
