# Supplementary material for: Wealth, health, and happiness: An inverse story of the Easterlin Paradox in China
Source: PLoS One. 2026 Mar 12;21(3):e0342445. doi: 10.1371/journal.pone.0342445 (PMC12981521; doi:10.1371/journal.pone.0342445)
Supplement: Attachment — Submitted filename: CommentsR2.docx [file pone.0342445.s005.docx]

**Comments to R2**

The manuscript was overall adequately revised, and unclear parts were mostly clarified. I also see that the authors have made substantial effort in language editing.

However, I have one major concern which may affect the entire conclusion and credibility of the study. Below, I explain the major concern, followed by minor comments.

**Major concern**

1. My major concern is the 2SLS estimations. Well, the estimations that the authors call 2SLS but actually seems to be two-step OLS mimicking 2SLS. I initially asked the authors why the sample size in column 1 of Table 4, one of the two first-stages, is just 4,224, while the sample size of the second-stage result is 22,199. The author replied that the column 1 was done at the city-level while others were done at the individual level, implying that each equation was separately estimated. Based on this reply, while the authors call it 2SLS, what the authors actually did seems to be as follows. The second stage has two endogenous regressors (SO2 and wage, denoted by P and R). The authors first estimated the first stages by OLS, using IVs, and obtained the hat-values. Then they plugged the hat-values of the endogenous regressors into the second stage and estimated the second stage again by OLS. Although this is the idea that its name, two-stage least square, implies, the manual implementation like above is a classic mistake because the standard errors are not accurately estimated. The same approach was used in Tables 8 and 11, where the samples were divided by years, and in Tables 13–16, where heterogeneity is explored. Indeed, in the manuscript pp 26–27, these procedures were described, although the authors state “2SLS” but do not mention that they used OLS actually.
2. The problem of this manual procedure mimicking 2SLS by OLS is that the standard errors are not accurately calculated (see e.g., Angrist and Pischke, Mostly Harmless Econometrics, Ch. 4.6 and Wooldridge, Econometric Analysis of Cross Section and Panel Data, Ch. 5.1). Almost always the standard errors are underestimated, making the coefficient of the endogenous regressor overly significant. The authors should use a command for 2SLS, such as ivregress and ivreg2—you are using stata, right? I wonder if the coefficients of endogenous regressors, particularly that of SO2, remain significant after you properly conduct 2SLS with these commands.
3. The authors seem to have a concern that SO2 variable is only at city level and the first stage for SO2 should not be done at individual level, which is the reason that they tried the mimicked 2SLS method. But this is not a concern at all. The authors can simply use ivreg or ivreg2, conducting the first stage for SO2 also at individual level.
4. In addition, applying this procedure to the heterogeneity analyses (column 3 of Tables 13–16), the authors seem to make a mistake of forbidden regression. I take Table 13 as an example, but the same concerns apply to all other tables. Although the authors do not use the terms “2SLS” and “IV” in the explanations of the equations (11)–(13), the method is actually the same mimicked 2SLS conducted by OLS. There are for endogenous regressors, P, P*developed, R, and R*developed. The authors seemingly used the same P-hat and R-hat as above. And then they interacted P-hat and R-hat with the “developed” dummy variable, and plugged P-hat, R-hat, P-hat*developed and R-hat*developed into the second stage and ran OLS. This is a classic example of forbidden regression.
5. This inappropriate procedure may be the reason that none of the coefficients of the interaction terms, P*developed and R*developed (Table 13) to P*educated and R*educated (Table 16) are significant.
6. Instead, if there are four endogenous regressors, P, P*developed, R, and R*developed, then there must be four first stages, where each endogenous variable is the dependent variable. As the number of IVs are not enough if only Day, Wind, and Avgwage are used as IVs, the authors may use Day*developed, Wind*developed, and R*developed as additional IVs. And these procedures should be done by ivregress, ivreg2 or other appropriate commands, not mimicking 2SLS by OLS.
7. If the interaction terms still do not provide significant coefficients even after appropriate procedure, you may simply drop these heterogeneity analyses.
8. The other major concerns I raised in the previous review were mostly clarified. However, this major concern is quite a large one. I was not sure at the moment of the previous review whether the explanation in the text is wrong (but the method is accurate) or the method itself has a problem, but it now turns out that the method has the problem of the mimicked-2SLS and forbidden regression.
9. Appropriate re-estimations may affect the significance of the coefficients. In particular, the coefficient of SO2 on SWB in Table 4 is significant at a merely 10% level as of now, and if it remains significant is not clear. This coefficient is of particular importance because it is the main evidence for the inverse logic of the environmental explanation of Easterlin’s Paradox.
10. By the way, just in case the appropriate 2SLS does not provide a significant effect of SO2, I am not sure why the endogeneity of SO2 level is a concern in the first place. The authors only note that “*Since both air quality and income might be correlated with factors which also influence SWB, we apply a series of instrumental variables to deal with potential endogeneity issues* (p25)” and do not argue what kind of unobservable factors confounds the relationship. But is there really any major factor that still affects both SWB and SO2 even after controlling for individual and household characteristics, city FE, and region-year FE? So, one possibility, if the appropriate 2SLS does not provide a significant effect, would be to treat SO2 as exogenous regressor, assuming that potential confounding factors are controlled by FEs. Indeed, Levinson (2012) uses IV only for income and treats pollution as exogenous. I do not know if this approach provides a desired result, but it is better than two-step OLS mimicking 2SLS.

Minor comments

11. The sample size must be provided in Tables 7, 8, 10, 11, 13–16. In addition, the author should explain what variables are used but omitted in these tables. As for Table 7, I commented before that the demonstration of YES rows and sample sizes were confusing, but I did not recommend you to completely remove them. You may attach notes below the table explaining the sample sizes and the control variables that were used but omitted. You explained these things in text, but it should also be clarified in tables.
12. Table 6 (effects of TCZ on health) and Table 7 (effects of health on SWB) are currently demonstrated in the subsection “Robustness Checks”, but they are not robustness checks. So, you may set up an additional subsection entitled “Mechanisms” or “Health Channel” or whatever and demonstrate them after Table 12—this is not mandatory, however.
13. Regarding the heterogeneity analyses, despite the insignificant differences between groups, the authors describe as if there are differences. For example, regarding the heterogeneity with respect to initial pollution (Table 14), while the authors state that “*the effects of the policy shock are not significantly different across high- and low-pollution cities*,” they also state that “*Residents in high pollution cities exhibit a lower WTP for pollution reduction*” (both p45). The authors also state in the conclusion that “*in terms of initial pollution level, it is stronger when the affected city has higher initial pollution level (p49)”.* Well, the WTP differs slightly (287 vs. 337), but this difference reflects the insignificant coefficients of interaction terms—or, in other words, the insignificant coefficients of interaction terms were treated as if they were significantly different from zero. Thus, it is unfair to conclude “*stronger when the affected city has higher initial pollution level*” as in the conclusion. The same applies to other heterogeneity analyses—although the results themselves may change if you appropriately avoid the methodological problems pointed out above.
14. Capitalization: Some table headings are capitalized (e.g., Table 2. The Impact of Regulation on SO2) whereas some others are not (e.g., Table 4. The relationship between income, air quality and SWB). Be consistent.
15. In addition, although I acknowledge that the authors made significant language editing, further language editing may be needed. For example, there is an incomplete sentence in pp11-12, “*With pooled cross-sectional data covering 214 cities in 22 provinces of China over the years 2002-2013. This dataset matches air pollution and…*”
