# Supplementary material for: Wealth, health, and happiness: An inverse story of the Easterlin Paradox in China
Source: PLoS One. 2026 Mar 12;21(3):e0342445. doi: 10.1371/journal.pone.0342445 (PMC12981521; doi:10.1371/journal.pone.0342445)
Supplement: Attachment — Submitted filename: Response_to_Reviewers_auresp_3.docx [file pone.0342445.s006.docx]

Dear Editor,

Thank you for your email of 23 June 2025 concerning our manuscript “Wealth, Health, and Happiness: An Inverse Story of the Easterlin Paradox in China” (PONE-D-23-32960). We are extremely grateful for the opportunity to revise our manuscript based on the reviewers' constructive feedback and your guidance. We have revised the paper according to the reviewer’s suggestions and have marked the changes in red.

The major revisions are summarized as follows:

1. The ivreg2 command in Stata, which is used for instrumental variable estimation, was employed to estimate the equation for calculating WTP. Considering the relative exogeneity of air pollution, we only used instrumental variables for personal wages. The regression results have been updated in Table 4.
2. A careful review of the data used was conducted, and the source of SO₂ data was replaced. The original data, derived from satellite data inversion, may have low accuracy. Therefore, based on the grid data from EDGAR v8.0, we recalculated city-level SO₂ emissions. This data has a finer scale and is more authoritative.

With this letter, we have resubmitted our manuscript which we would like to be considered for publication in PLOS ONE. In order to facilitate the review of our revisions, we are attaching a detailed, comment by comment response to the reviewer’s concerns.

If you need anything else, please do not hesitate to contact us.

Kind regards,

All authors

Response of Authors to the Comments of Reviewer #1

“Wealth, Health, and Happiness: An Inverse Story of the Easterlin Paradox in China”

| **Reviewer’s Comments** | **Authors’ Response** |
| --- | --- |
| Comments to the Author  GENERAL COMMENTS  I appreciate the efforts made by the authors to address my previous comments. I feel that this version has been well improved. Yet, I still have some questions and suggestions for the authors. | Thank you very much for your encouraging comments and constructive suggestions. We greatly appreciate your recognition of our previous efforts. We have revised the manuscript carefully in response to your remaining comments. We hope you find the revisions satisfactory. |
| MAJOR COMMENTS   1. 1) I still don’t understand how did you conduct the IV estimation. Did you estimate Eq(3) and Eq(4) separately to predict P and R, or you estimate them all together? Since the set of control variables are different for Eq (3) and Eq (5), it may raise the concern of forbidden regression. Moreover, you should also report the results of weak instruments test and over-identification test to justify the validity of your instruments. | Thank you for your insightful comment on the instrumental variable (IV) estimation approach, we apologize for the ambiguity in our previous submission that led to your confusion. To address your questions directly:   1) **IV estimation method**: Initially, we estimated Eq. (3) and Eq. (4) separately to predict Pollution and Wage. However, we recognize that the different sets of control variables across Eq. (3) and Eq. (5) may may lead to issues related to forbidden regressions, mainly because the previous implementation relied on manually generated fitted values. To address this properly, we now use ivreg2, which automatically performs the correct two-stage least squares estimation and computes valid standard errors. 2) **Weak instrument test**: We have supplemented the weak instrument test results in Table 4, including the Cragg-Donald Wald F-statistic (value: 165.782) and the Kleibergen-Paap rk Wald F statistic (value: 140.884). Since these F-statistics far exceed the conventional critical values, the test confirms that our instrument is not weak, validating its relevance. 3) **Over-identification test:** In the updated model, we adopt a just-identified specification (one instrumental variable for one endogenous variable: only personal wage is treated as endogenous, while air pollution is treated as relatively exogenous). Over-identification tests (such as the Sargan-Hansen test) require more instruments than endogenous variables to assess instrument exogeneity. As our model is just-identified, this test is not applicable. We have clarified this model setting in the manuscript (p. 26-27) to avoid confusion.   Thank you again for your constructive feedback-this revision has significantly enhanced the methodological rigor and transparency of our work. |
| 2) Is it possible to control for stricter fixed effect such as individual fixed effects in Eq (5)? The SWB equation is the base of all your calculation, which is very important to support your conclusion. Therefore, I would like to see more robustness checks about the SWB equation. | Thank you for your valuable suggestion regarding the robustness of the SWB equation (formerly Eq. 5, now Eq. 4). We fully agree that this equation is foundational to our conclusions.  Regarding the feasibility of adding individual fixed effects: We regret that this is not available for Eq. (4), as the estimation relies on CHIP data, which is pooled cross-sectional data (rather than panel data). Individual fixed effects control for time-invariant unobserved heterogeneity of the same individuals over multiple periods, which requires repeated observations of the same individuals across different time waves.  Although individual fixed effects cannot be used due to the pooled cross-sectional nature of the CHIP data, we conducted several robustness checks-such as year-interacted specifications and geographic restrictions-to verify that the estimates from Eq. (4) remain stable.  The results of these robustness checks confirm that our core findings from Eq. (4) remain consistent and reliable. Thank you again for helping us strengthen the rigor of our analysis. |
| 3) There are inconsistences in the terminology in the paper. For example, In Eq(2), the dependent variable is defined as the average monthly income of individual. However, in Table 3 and 4, the income becomes wage. You should keep the same terminology throughout the paper. | Thank you for pointing out the terminology inconsistency-this is a key detail for clarity, and we appreciate your careful review.  We note that the variable in question (covering annual wage, bonus, and other income from the job) is more accurately defined as income (rather than narrow wage). To resolve the inconsistency:  We have revised all instances of wage in Tables 3, 4, and the corresponding text to R (consistent with the definition in Eq. 2: average monthly income of individual).  We have double-checked the entire manuscript to ensure this terminology is applied uniformly across equations, tables, and descriptions.  This revision aligns the terminology with the actual scope of the variable and eliminates confusion. Thank you again for your meticulous feedback. |
| 4) To improve the reading experience of the paper, I would suggest to put forward the analysis of SWB before the TCZ analysis. You should first estimate the willingness to pay for air pollution reduction, then proceed to estimate the reduction of air pollution by TCZ policy and calculate monetary value of related improvement of wellbeing. | Thank you for your thoughtful suggestion regarding the manuscript’s structure. We truly value your input on improving the reading experience.  After carefully considering your suggestion to present the SWB analysis before the TCZ analysis, we respectfully propose to maintain the current structure for the following reasons, which we believe best serve the logical flow of the research:  The core objective of our study is to examine the causal mechanism of how the TCZ policy (an environmental intervention) affects subjective well-being (SWB). The current organization aligns strictly with this causal chain:  1) Policy Evaluation: We first verify that the TCZ policy effectively reduces air pollution and income (TCZ analysis).  2) Impact Mechanism: We then estimate how this reduction in air pollution and income impacts SWB (SWB equation).  3) Valuation: finally, based on these links, we calculate the Willingness to Pay (WTP) for pollution reduction to quantify the welfare effect.  This order is designed to guide readers naturally from the policy intervention → environmental outcome  → well-being impact → economic valuation. Placing the SWB analysis first might disrupt this narrative of evaluating a specific policy's welfare effect.  We hope this explanation clarifies the rationale behind our structural choice, and we appreciate your understanding. Thank you again for your valuable feedback. |
| 5) Table 4 is very important, and have you included any control variables? It is not clear in the table. And all tables should have more detailed notes to make it self-readable. | Thank you for your constructive suggestion to improve the clarity of the tables, we fully agree that detailed notes and transparent variable reporting are critical for readability. In response to your concern:  For Table 4: We have revised the table to explicitly include the regression results of control variables (which were previously omitted for brevity) and supplemented the note to clarify this. The updated note for Table 4 now reads: Table 4 reports the estimation results of Equation (3) and Equation (4). Column (1) presents the first-stage IV regression corresponding to Equation (3). The Cragg-Donald Wald F statistic (165.782) and the Kleibergen-Paap rk Wald F statistic (140.884) both exceed conventional thresholds, indicating that the instrument is strongly identified. Column (2) reports the second-stage IV estimates corresponding to Equation (4). (We have also adjusted the table layout to display these control variable coefficients.)  For all tables: We have added detailed self-explanatory notes to each table, which clarify:  1) The corresponding equation for each table;  2) The specification of each column (e.g., time function, fixed effects);  3) Key statistical details (e.g., robust standard errors, significance levels);  4) Supplementary information (e.g., WTP calculation basis in Table 8).  These revisions ensure that each table is self-contained and easy to interpret without relying on the main text. We have checked all tables to confirm the notes align with the results. Thank you again for your meticulous feedback. |

Response of Authors to the Comments of Reviewer #2

“Wealth, Health, and Happiness: An Inverse Story of the Easterlin Paradox in China”

| **Reviewer’s Comments** | **Authors’ Response** |
| --- | --- |
| Comments to the Author  GENERAL COMMENTS  The manuscript was overall adequately revised, and unclear parts were mostly clarified. I also see that the authors have made substantial effort in language editing.  However, I have one major concern which may affect the entire conclusion and credibility of the study. Below, I explain the major concern, followed by minor comments. | Thank you very much for your positive overall evaluation of our revised manuscript and for acknowledging our efforts in clarification and language editing. We greatly appreciate the time and care you have devoted to reviewing our work.  We also recognize the major concern you raised regarding the 2SLS estimation procedure. We take this issue very seriously. Below, we address your concern point-by-point and detail the corresponding revisions made to the manuscript. |
| **Major concern**  1) My major concern is the 2SLS estimations. Well, the estimations that the authors call 2SLS but actually seems to be two-step OLS mimicking 2SLS. I initially asked the authors why the sample size in column 1 of Table 4, one of the two first-stages, is just 4,224, while the sample size of the second-stage result is 22,199. The author replied that the column 1 was done at the city-level while others were done at the individual level, implying that each equation was separately estimated. Based on this reply, while the authors call it 2SLS, what the authors actually did seems to be as follows. The second stage has two endogenous regressors (SO2 and wage, denoted by P and R). The authors first estimated the first stages by OLS, using IVs, and obtained the hat-values. Then they plugged the hat-values of the endogenous regressors into the second stage and estimated the second stage again by OLS. Although this is the idea that its name, two-stage least square, implies, the manual implementation like above is a classic mistake because the standard errors are not accurately estimated. The same approach was used in Tables 8 and 11, where the samples were divided by years, and in Tables 13–16, where heterogeneity is explored. Indeed, in the manuscript pp 26–27, these procedures were described, although the authors state “2SLS” but do not mention that they used OLS actually.  2) The problem of this manual procedure mimicking 2SLS by OLS is that the standard errors are not accurately calculated (see e.g., Angrist and Pischke, Mostly Harmless Econometrics, Ch. 4.6 and Wooldridge, Econometric Analysis of Cross Section and Panel Data, Ch. 5.1). Almost always the standard errors are underestimated, making the coefficient of the endogenous regressor overly significant. The authors should use a command for 2SLS, such as ivregress and ivreg2—you are using stata, right? I wonder if the coefficients of endogenous regressors, particularly that of SO2, remain significant after you properly conduct 2SLS with these commands.  3) The authors seem to have a concern that SO2 variable is only at city level and the first stage for SO2 should not be done at individual level, which is the reason that they tried the mimicked 2SLS method. But this is not a concern at all. The authors can simply use ivreg or ivreg2, conducting the first stage for SO2 also at individual level.  4) In addition, applying this procedure to the heterogeneity analyses (column 3 of Tables 13-16), the authors seem to make a mistake of forbidden regression. I take Table 13 as an example, but the same concerns apply to all other tables. Although the authors do not use the terms “2SLS” and “IV” in the explanations of the equations (11)-(13), the method is actually the same mimicked 2SLS conducted by OLS. There are for endogenous regressors, P, P*developed, R, and R*developed. The authors seemingly used the same P-hat and R-hat as above. And then they interacted P-hat and R-hat with the “developed” dummy variable, and plugged P-hat, R-hat, P-hat*developed and R-hat*developed into the second stage and ran OLS. This is a classic example of forbidden regression.  5) This inappropriate procedure may be the reason that none of the coefficients of the interaction terms, P*developed and R*developed (Table 13) to P*educated and R*educated (Table 16) are significant.  6) Instead, if there are four endogenous regressors, P, P*developed, R, and R*developed, then there must be four first stages, where each endogenous variable is the dependent variable. As the number of IVs are not enough if only Day, Wind, and Avgwage are used as IVs, the authors may use Day*developed, Wind*developed, and R*developed as additional IVs. And these procedures should be done by ivregress, ivreg2 or other appropriate commands, not mimicking 2SLS by OLS.  7) If the interaction terms still do not provide significant coefficients even after appropriate procedure, you may simply drop these heterogeneity analyses.  8) The other major concerns I raised in the previous review were mostly clarified. However, this major concern is quite a large one. I was not sure at the moment of the previous review whether the explanation in the text is wrong (but the method is accurate) or the method itself has a problem, but it now turns out that the method has the problem of the mimicked-2SLS and forbidden regression.  9) Appropriate re-estimations may affect the significance of the coefficients. In particular, the coefficient of SO2 on SWB in Table 4 is significant at a merely 10% level as of now, and if it remains significant is not clear. This coefficient is of particular importance because it is the main evidence for the inverse logic of the environmental explanation of Easterlin’s Paradox.  10) By the way, just in case the appropriate 2SLS does not provide a significant effect of SO2, I am not sure why the endogeneity of SO2 level is a concern in the first place. The authors only note that “Since both air quality and income might be correlated with factors which also influence SWB, we apply a series of instrumental variables to deal with potential endogeneity issues (p25)” and do not argue what kind of unobservable factors confounds the relationship. But is there really any major factor that still affects both SWB and SO2 even after controlling for individual and household characteristics, city FE, and region-year FE? So, one possibility, if the appropriate 2SLS does not provide a significant effect, would be to treat SO2 as exogenous regressor, assuming that potential confounding factors are controlled by FEs. Indeed, Levinson (2012) uses IV only for income and treats pollution as exogenous. I do not know if this approach provides a desired result, but it is better than two-step OLS mimicking 2SLS. | Thank you very much for your careful and constructive comments on our IV strategy. We truly appreciate the precision with which you identified the issues in our previous implementation. Following your suggestions, we have undertaken a full revision of all empirical estimations involving instrumental variables. Below, we provide a point-by-point response.  1) **Correction of the 2SLS implementation**  We sincerely appreciate you pointing out the methodological flaw in our previous manual “two-step OLS” procedure. As advised, we have re-estimated all specifications that involve instrumental variables (Tables 4, 8, 10 and the heterogeneity tables) using ivreg2 in Stata, which correctly estimates the first and second stages jointly and provides valid standard errors.  All results previously based on manual 2-step OLS have been removed and replaced with the correct 2SLS results (Tables 4, 8, and 10). Although the re-estimated coefficients differ slightly from the previous results-as is expected when correcting the IV procedure-the overall qualitative conclusions of the study remain unchanged.  2) **The coefficient of SO_2_ and its treatment as exogenous**  Following your concerns in Points (2), (9), and (10): When we treated both income and SO₂ as endogenous in ivreg2, the SO₂ coefficient became statistically insignificant.  This result, consistent with your expectation, suggests that the previous significance was driven by the misestimated standard errors in the manual procedure.  You also raised an important conceptual point: given the extensive controls (household, city FE, region-year FE), the endogeneity of SO₂ may not be a major concern, and previous literature (e.g., Levinson 2012) also treats pollution as exogenous while instrumenting income.  Following this reasoning, we re-estimated the main model by treating SO₂ as exogenous and instrumenting only income. Under this specification, the SO₂ coefficient remains statistically significant (at the 10% level) with a magnitude consistent with the literature. This specification is now adopted as our baseline, and the justification has been added to the text.  3) **City-level SO_2_ in individual-level 2SLS**  Thank you for clarifying that city-level variables can be used directly in individual-level IV estimation. We have fully implemented your suggestion and now estimate all models at the individual level using ivreg2 without any manual aggregation or separate first-stage regressions.  4) **Correction of forbidden regression in heterogeneity analyses**  As you noted in Points (4)–(6), our previous heterogeneity analyses incorrectly interacted fitted values with subgroup dummies, resulting in forbidden regression. Following your suggestion, we re-estimated the heterogeneity specifications using the correct approach.  After re-estimating the models using the appropriate IV procedures, none of the interaction terms remained statistically significant. Since these results do not offer additional insights beyond the baseline findings, and in order to maintain focus and clarity, we decided to remove the heterogeneity section in the revised manuscript, as you suggested in point7.  5) **Summary of revisions**  All IV-related estimations have been re-run using ivreg2.  The baseline specification now treats SO₂ as exogenous and instruments only income.  The heterogeneity section (Tables 13-16) has been removed after correct re-estimation.  Your comments greatly improved the methodological rigor and internal validity of our study. We are sincerely grateful for your guidance. |
| 2. Minor comments  1) The sample size must be provided in Tables 7, 8, 10, 11, 13–16. In addition, the author should explain what variables are used but omitted in these tables. As for Table 7, I commented before that the demonstration of YES rows and sample sizes were confusing, but I did not recommend you to completely remove them. You may attach notes below the table explaining the sample sizes and the control variables that were used but omitted. You explained these things in text, but it should also be clarified in tables. | Thank you for your meticulous feedback on the tables. We fully agree that clear reporting of sample sizes and control variables is critical for transparency and readability. We have revised the relevant tables as follows:   1) Supplement of sample sizes   We have added the sample size (Observations = 23,187) to Tables 7 (Tables 13-16 have been removed due to the heterogeneity analysis revision, as explained in our previous response).   2) Clarification of included but omitted variables   For Table 7, We have added a dedicated row section in the table to list all control variables and fixed effects, with YES indicating inclusion (as you noted: Individual Control, Family Control, Weather Control, Year FE, City FE, Province-by-year FE). We have also updated the note to link these to the omitted variables: All control variables marked YES in the table are included in the regression but their coefficients are omitted for brevity.  For Tables 8, We have added the note: Table 8 reports the year-by-year results of Equation (4), with Columns (1)-(4) corresponding to different years (total observations = 23,187). For brevity, we only present the coefficients of SO_2_, Ln(R). The sample mean of income ($\bar{R}_{CHIP}$) is directly calculated from the corresponding CHIP subsample, while the implied WTP is computed using the estimated ratio $\mid\frac{\hat{\beta}_{4}}{\hat{\gamma}_{4}}\mid$and $\bar{R}_{CHIP}$. All variables included in Equation (4) are included in the regressions. Robust standard errors are reported in parentheses (*** p < 0.01, ** p < 0.05, * p < 0.1).  For Table 10, We have added the note: Table 10 reports the year-by-year estimates of Equation (4) using the CHIP dataset restricted to provinces also covered by the CHNS (total observations = 9,413). Columns (1)-(4) present results for 2002, 2007, 2008, and 2013, respectively. As in Table 8, we report only the coefficients of SO_2_, Ln(R). The sample mean of income ($\bar{R}_{CHIP}$) is directly calculated from the corresponding CHIP subsample, while the implied WTP is computed using the estimated ratio $\mid\hat{\beta}_{4}/\hat{\gamma}_{4}\mid$and $\bar{R}_{CHIP}$. All variables included in Equation (4) are included in the regressions. Robust standard errors are reported in parentheses (*** p < 0.01, ** p < 0.05, * p < 0.1).   3) Revision of Table 7’s note (aligning with supplements)   The updated note for Table 7 now reads: Table 7 presents the estimation results of Equation (9), reporting the marginal effects from the ordered logit model that measure the impact of individual health status on SWB. Column (1) shows the marginal effect of health2 in Equation (9), while Column (2) shows the marginal effect of health3. Column (3) reports the difference between (1) and (2). All control variables and fixed effects marked YES in the table are included in the regression but their coefficients are omitted for brevity. Robust standard errors are reported in parentheses (** p < 0.01, ** p < 0.05, * p < 0.1).  All revisions ensure that sample sizes and included (but omitted) variables are clearly stated in both the table layout and notes, eliminating the need for readers to refer to the main text. Thank you again for helping us improve the transparency of our results. |
| 2) Table 6 (effects of TCZ on health) and Table 7 (effects of health on SWB) are currently demonstrated in the subsection “Robustness Checks”, but they are not robustness checks. So, you may set up an additional subsection entitled “Mechanisms” or “Health Channel” or whatever and demonstrate them after Table 12-this is not mandatory, however. | Thank you very much for your thoughtful suggestion regarding the organization of the manuscript. We truly appreciate your perspective on improving the logical flow.  After careful consideration, we have decided to retain Tables 6 and 7 in the “Robustness Checks” subsection, while adding a clarification to avoid possible confusion. Our rationale is as follows:  Although Tables 6 and 7 indeed document the health transmission channel (TCZ → health → SWB), they are included in the robustness section because we re-estimate both relationships using alternative model specifications. The purpose is to verify that the key links in the mechanism remain stable under different setups, which fits the objective of the robustness section-namely, to validate the reliability of all core results.  To address your comment, we have added a clarifying introductory sentence in the robustness subsection (p. 37):  Third, our conceptual framework suggests that the TCZ policy may improve SWB by reducing air pollution and thereby enhancing health. Since our baseline results show a significant reduction in pollution, we next test the robustness of this health transmission channel. We re-estimate the effect of the TCZ policy on health (Table 6) and the effect of health on SWB (Table 7) under alternative specifications.  This clarification makes explicit that Tables 6 and 7 serve both to illustrate the mechanism and to confirm its robustness. We appreciate your flexibility in noting that reorganization is not mandatory, and we believe that the revised structure with added explanation ensures clarity while maintaining coherence.  Thank you again for your valuable and constructive feedback. |
| 3) Regarding the heterogeneity analyses, despite the insignificant differences between groups, the authors describe as if there are differences. For example, regarding the heterogeneity with respect to initial pollution (Table 14), while the authors state that “the effects of the policy shock are not significantly different across high- and low-pollution cities,” they also state that “Residents in high pollution cities exhibit a lower WTP for pollution reduction” (both p45). The authors also state in the conclusion that “in terms of initial pollution level, it is stronger when the affected city has higher initial pollution level (p49)”. Well, the WTP differs slightly (287 vs. 337), but this difference reflects the insignificant coefficients of interaction terms-or, in other words, the insignificant coefficients of interaction terms were treated as if they were significantly different from zero. Thus, it is unfair to conclude “stronger when the affected city has higher initial pollution level” as in the conclusion. The same applies to other heterogeneity analyses-although the results themselves may change if you appropriately avoid the methodological problems pointed out above. | Thank you very much for your careful and constructive comments regarding the heterogeneity analyses. We fully acknowledge that our previous descriptions were inappropriate, as they implied group differences that were not supported by statistically significant interaction terms.  In response, and also in light of the methodological issues you highlighted earlier (particularly the forbidden regression problem in treating interaction terms involving an endogenous variable), we have decided to remove the entire heterogeneity analysis section from the revised manuscript. This choice is motivated by two considerations:  The interaction coefficients are statistically insignificant, meaning that the data do not support meaningful group differences.  The methodological limitations imply that the previous heterogeneity estimates may not be reliable.  Correspondingly, we have also deleted all descriptive statements in the discussion and conclusion sections that suggested group differences-including the previous claims such as “residents in high-pollution cities exhibit a lower WTP” and “the effect is stronger in cities with higher initial pollution levels.”  We sincerely appreciate your pointing out this issue. Your comments have helped us ensure that all claims in the manuscript strictly align with the statistical evidence and maintain the integrity of our conclusions. |
| 4) Capitalization: Some table headings are capitalized (e.g., Table 2. The Impact of Regulation on SO2) whereas some others are not (e.g., Table 4. The relationship between income, air quality and SWB). Be consistent. | Thank you for pointing out the inconsistency in the capitalization of table headings. We appreciate your careful attention to detail.  To address this, we have standardized the capitalization format across all table headings in the manuscript. Specifically, we have adopted sentence case-that is, only the first word and proper nouns are capitalized-consistent.  For example: We revised Table 2’s heading from “The Impact of Regulation on SO2” to “The impact of regulation on SO2”  All other table headings have been updated to follow the same sentence-case format.  Thank you again for your helpful suggestion, which improves the consistency and professionalism of the manuscript. |
| 5) In addition, although I acknowledge that the authors made significant language editing, further language editing may be needed. For example, there is an incomplete sentence in pp11-12, “*With pooled cross-sectional data covering 214 cities in 22 provinces of China over the years 2002-2013. This dataset matches air pollution and…* | Thank you for your meticulous feedback on the manuscript’s language. We appreciate your careful reading and for pointing out the incomplete sentence.   1) Correction of the incomplete sentence (pp. 11–12):   You are absolutely right that the original expression was a sentence fragment. We have now revised and integrated it into a complete sentence. The corrected version reads:  “We use pooled cross-sectional data covering 214 cities across 22 provinces from 2002 to 2013, matching city-level pollution measures and economic variables to individual respondents. This structure allows us to capture more comprehensively how environmental conditions affect SWB.”  2) Further language editing:  In response to your suggestion, we conducted an additional round of language polishing, focusing on grammar, clarity, and academic tone. Several expressions were revised to improve precision and readability. For example:   - p.3:   That is, since income growth over time is usually accompanied by industrialization and pollution…  → The idea is that long-run income growth is typically accompanied by industrialization and pollution, which impose welfare costs not captured by income measures…   - p.8   this phenomenon, which is the named the Easterlin paradox,  → this phenomenon, known as the Easterlin paradox,   - p.13   There were growing worries that acid rain could damaging ecosystems…  → There were growing concerns that acid rain could damage ecosystems....  These revisions, along with other minor grammatical corrections, have improved the linguistic quality of the manuscript. We sincerely appreciate your feedback, which has greatly enhanced the clarity of the paper. |
