# Supplementary material for: Wealth, health, and happiness: An inverse story of the Easterlin Paradox in China
Source: PLoS One. 2026 Mar 12;21(3):e0342445. doi: 10.1371/journal.pone.0342445 (PMC12981521; doi:10.1371/journal.pone.0342445)
Supplement: Attachment — Submitted filename: Response_to_Reviewers_auresp_4.docx [file pone.0342445.s007.docx]

Dear Editor,

Thank you for your email dated December 20, 2025 regarding our manuscript, “Wealth, Health, and Happiness: An Inverse Story of the Easterlin Paradox in China” (PONE-D-23-32960). We are grateful for the opportunity to revise our manuscript in response to the reviewers’ constructive comments and your guidance.

We have carefully revised the manuscript to address all concerns raised during the review process. A detailed, point-by-point response to the reviewers’ comments is provided in the accompanying Response to Reviewers document. We have also submitted both a clean revised manuscript and a version with tracked changes to facilitate evaluation.

We sincerely appreciate the time and effort invested by the editor and reviewers, which have greatly helped to strengthen the quality of our work. We hope that the revised manuscript now meets the publication standards of PLOS ONE and would be grateful for your further consideration.

Please do not hesitate to contact us if any additional information is required.

Kind regards,

All authors

Response of Authors to the Comments of Reviewer #1

“Wealth, Health, and Happiness: An Inverse Story of the Easterlin Paradox in China”

| **Reviewer’s Comments** | **Authors’ Response** |
| --- | --- |
| Comments to the Author  GENERAL COMMENTS  The paper is well revised and the authors have addressed all my comments, I think the paper is ready for publication. | We sincerely thank the reviewer for the positive evaluation of our manuscript. We greatly appreciate the time and thoughtful comments provided throughout the review process. |

Response of Authors to the Comments of Reviewer #2

“Wealth, Health, and Happiness: An Inverse Story of the Easterlin Paradox in China”

| **Reviewer’s Comments** | **Authors’ Response** |
| --- | --- |
| Comments to the Author  GENERAL COMMENTS  The authors addressed the methodological concerns. The overall methodologies are fair.  I suggested in the previous review that the authors may quit instrumenting the SO2 level. The authors previously instrumented it (but in an inappropriate method mimicking 2SLS). The authors admitted that, after using a correct 2SLS method, the significance of the coefficient of SO2 on SWB disappeared, and they chose not to instrument SO2. Certainly, a method that appropriately account for the endogeneity of air pollution is preferrable. However, I at least believe that a method treating SO2 level as exogenous is better than an inappropriate method that wrongly provides a seemingly good result.  The authors also improved the interpretation of their results and removed overstating claims, which I positively evaluate.  Meanwhile, the authors also made various other methodological changes that neither I nor the other reviewer suggested. Making such changes itself is fine if it improves the quality of the study. However, the appropriateness of some of these changes is questionable at times. Below are the main concerns. | We sincerely thank the reviewer for the careful evaluation of our revised manuscript and for the constructive feedback provided in the previous and current rounds of review.  Regarding the treatment of SO₂, we agree with your assessment that an appropriately specified model treating SO₂ as exogenous is preferable to an incorrect IV approach, and we have revised the analysis accordingly.  We also acknowledge your concern regarding the additional methodological changes introduced in this revision. These changes were intended to improve the analysis, and we address each of the specific points you raise below. |
| **Major concern**  1) What is the reason that the sample size changed from the previous version? The observation size for city-level estimations increased from 680 to 745 (Table 2), that in the CHNS based estimations decreased from 27,561 to 11,088 (Table 3) and that in the CHIP based estimations slightly increased from 22,199 to 23,187. The change of the CHNS based estimations is particularly drastic. What is the reason? Did you change the sample criteria? Or, considering the errors having occurred in the previous manuscript, are the sample sizes correct?  . | We sincerely thank the reviewer for raising this important question regarding the changes in sample sizes across revisions. Below, we clarify the reasons for each change and confirm that the current samples are correctly constructed and consistent with the revised empirical specifications.   - City-level estimations (Table 2: 680 → 745).   In the revised version, we expanded the city-level sample by incorporating additional cities from Heilongjiang Province that were inadvertently omitted in the previous data-matching procedure. This correction improves regional coverage and results in a more complete and representative city-level dataset.   - CHNS-based estimations (Table 3: 27,561 → 11,088).   In the previous version, we imputed income using the employment status to increase sample size (e.g., assigning zero income to non-employed individuals). In the revised analysis, we use only income values directly reported in the CHNS and do not impute income based on employment status, which reduces the sample size but improves measurement accuracy.   - CHIP-based estimations (Table 4: 22,199 → 23,187).   In the earlier version, observations with zero income were dropped mechanically due to the logarithmic transformation of income. In the revised version, we retain these observations by adding a small constant (0.0001) to income values prior to taking logarithms, which allows us to preserve zero-income observations without materially affecting the estimation results.  Overall, these changes reflect improved data handling and greater internal consistency between variable construction and the empirical specifications. The reported sample sizes follow directly from the updated data construction and processing procedures described above. |
| 2) The addition of P*f(t) in Tables 2, 3 and 6 is questionable. P is the pre-TCZ SO2 emission. This is particularly questionable in Table 2 in which the dependent variable is the SO2 level. The authors previously did not add such a term and used only year FE, city FE, and province-by-year FE, which worked. What is the reason that the authors made this change? The authors explain that they added this term to “control for differential pre-policy trends associated with initial pollution levels (p24)” but it was not necessary in the previous version. Even if the authors try this method, the authors should also show the results in which P*f(t) is not added. In addition, the authors show the parallel trends in Figures 3 and 4, meaning that the differential pre-policy trends are not really a concern. Therefore, the authors should try a model without P*f(t) first and then may try models with P*f(t) as a robustness check. | We sincerely thank the reviewer for this thoughtful and constructive comment regarding the inclusion of the interaction term $P\times f(t)$, where $P$denotes pre-policy SO₂ emissions.  We agree that in many DID applications, models with only unit and time fixed effects can be sufficient when the parallel trends assumption holds. However, in the case of the TCZ policy, city selection was explicitly based on pre-policy pollution severity, which raises concerns about heterogeneous pre-treatment trends across cities with different initial pollution levels.  In this sense, we follow the most recent DID practices (e.g., Li et al., 2016; Liu et al., 2025), and include $P\times f(t)$ to flexibly control for potential trend heterogeneity linked to initial SO₂ levels.  In response to your kind suggestion, we also added the estimation results and parallel trend tests from the specifications without $P\times f(t)$ in Appendix A (Tables A1–A2 and Figures A1–A2), and added a footnote in the main text to explicitly inform readers of these alternative specifications. In these results, we find that when $P\times f(t)$ is excluded, the pre-treatment trend for the logarithm of income is not parallel, and the estimated effect of the TCZ policy on SO₂ is counterintuitively positive. In contrast, once $P\times f(t)$ is included, both problematic phenomena are addressed. We therefore view the inappropriate results in the $P\times f(t)$ excluded models as outcomes of unaccounted heterogeneous trends and recommend the results from the $P\times f(t)$ included models in the main text as credible policy effects.  We sincerely appreciate the reviewer’s constructive suggestion, which has helped us clarify and strengthen our empirical strategy. |
| 3) Why did the authors include individual FE for balancing test? It is obvious that the differences in individual and household characteristics disappears once you add individual FE, because it basically absorbs any time-invariant individual-level characteristics. And if you are using a panel dataset in a FE model, the sample balance is not a concern. This table is simply unnecessary. | We sincerely thank the reviewer for this insightful comment.  The purpose of this balancing test is not to assess sample balance in the conventional sense. Rather, following Tanaka (2015) and Wang et al. (2024), it serves as a supplementary diagnostic to examine whether the TCZ policy variable is systematically correlated with observable time-varying individual-, household-, and city-level characteristics, conditional on individual fixed effects and time controls. This provides additional descriptive evidence on the plausibility of “local randomness” with respect to time-varying covariates.  To streamline the presentation and avoid confusion, we have moved this table to the Appendix and clearly describe it as supplementary evidence. We are also happy to remove it entirely if the editor deems it unnecessary. |
